# Impact of *Helicobacter pylori* eradication on the gastric microbiome

**DOI:** 10.1186/s13099-021-00460-2

**Published:** 2021-10-13

**Authors:** Li-Qi Mao, Yan-Lin Zhou, Shuang-Shuang Wang, Lin Chen, Yue Hu, Lei-Min Yu, Jing-Ming Xu, Bin Lyu

**Affiliations:** 1 Department of Gastroenterology, The First Affiliated Hospital of Zhejiang Chinese Medical University, Hangzhou, China; 2 Department of Gastroenterology, The First People’s Hospital of Huzhou, The First Affiliated Hospital of Huzhou Teachers College, Huzhou, China; 3 Department of Gastroenterology, Taizhou Hospital of Zhejiang Province affiliated to Wenzhou Medical University, Taizhou, China; 4 Department of Gastroenterology, Guangxing Hospital Affiliated to Zhejiang Chinese Medical University, Hangzhou, China

**Keywords:** 16S rDNA gene sequencing, *Helicobacter pylori*, Eradication therapy, Gastric microflora, Atrophic gastritis

## Abstract

**Background:**

*Helicobacter pylori* (Hp) eradication has been used for many years. Yet, the impact of this eradication on the normal gastric microflora is not well understood. In this study, we explored the effect of eradication on the stomach microbial community and its recovery after successful Hp eradication.

**Methods:**

Among the 89 included patients, 23, 17, 40, and 9 were included in the Hp-negative, Hp-positive, successful eradication, and failed eradication groups, respectively. Four subgroups were further determined according to disease status (Hp-negative chronic gastritis [N-CG], Hp-negative atrophic gastritis [N-AG], successful-eradication chronic gastritis [SE-CG], and atrophic gastritis with successful eradication [SE-AG]). During the endoscopic examination, one piece of gastric mucosa tissue was obtained from the lesser curvature side of the gastric antrum and gastric corpus, respectively. In addition, 16S rDNA gene sequencing was used to analyze the gastric mucosal microbiome.

**Results:**

In the Hp-negative group, the gastric microbiota was dominated by five phyla: *Firmicutes*, *Proteobacteria*, *Actinobacteria*, *Bacteroidetes*, and *Fusobacteria*. After successfully eradicating Hp, the bacterial flora in the stomach recovered to a considerable extent. In the failed eradication group, the flora was similar to the flora in Hp-positive subjects based on the alpha and beta diversities. Among the groups, *Curvibacter* and *Acinetobacter* were enriched in the presence of Hp (i.e., failed eradication and Hp-positive groups), suggesting that these two genera could be used as biomarkers in the symbiotic flora in the presence of Hp. SE-CG was characterized by an increase in *Firmicutes* taxa and a decrease in *Proteobacteria* taxa compared with N-CG. SE-AG was characterized by a decrease in *Firmicutes* relative to N-AG. Finally, no differences were found in the pairwise comparisons of nitrate and nitrite reductase functions of the microflora among the four subgroups.

**Conclusions:**

After Hp infection, the diversity and relative abundance of gastric microflora were significantly decreased. Yet, gastric microbiota could be partially restored to the Hp-negative status after eradication. Still, this effect was incomplete and might contribute to the long-term risks.

**Supplementary Information:**

The online version contains supplementary material available at 10.1186/s13099-021-00460-2.

## Introduction

The gastrointestinal microbiome plays an important role in digestion, absorption, metabolism, immunity, and inhibition of pathogen colonization [1, 2]. The imbalance of its structure or function can lead to many diseases [1, 2].

*Helicobacter pylori* (Hp) is the most important and most studied bacterium of the stomach [3, 4]. Hp is a spiral-shaped Gram-negative bacterium transmitted through the fecal-oral route present in the gastrointestinal tract of more than half of people worldwide [5–7]. The worldwide prevalence of Hp infection is 44–49%; more specifically, it is 26–37% in North America, 35–47% in Europe, 45% in Asia, 57–79% in Africa, 60–63% in Latin America, and 24–48% in Oceania [8, 9]. In China, the prevalence of Hp infection is 52–62% [10, 11]. It is associated with various gastrointestinal diseases such as chronic gastritis, peptic ulcer, gastric mucosa-associated lymphoid tissue lymphoma, and gastric cancer [5–7, 12, 13]. Most patients acquire the infection during childhood [5–7]. Men appear to have slightly higher infection rates than women in adulthood, while in childhood sex ratio appears about even [5]. The risk factors include being socially disadvantaged, having a high number of siblings, and residing in or near endemic areas [5]. Adults and children infected with Hp can be asymptomatic or present with symptoms such as dyspepsia, epigastric abdominal pain, or signs of gastrointestinal bleeding. Smoking and chronic nonsteroidal anti-inflammatory drug (NSAID) use significantly increase the risk of peptic ulcer disease in those infected with Hp. Hp eradication reduces the incidence of gastric cancer, and this benefit becomes more pronounced with increasing age [14–16]. Currently, eradication aims to prevent the development of stomach cancer [17, 18].

The core of Hp eradication treatment is the acid-suppressive effect of proton pump inhibitors (PPIs) and the bactericidal effect of antibiotics [3, 4, 12, 14, 19–22], but antibiotic resistance is a growing concern [5, 6]. PPIs increase the gastric pH, while Hp prefers an acidic growth medium, and antibiotics exert a direct bactericidal effect [3, 4, 12, 14, 19–22]. The usual eradication regimens include bismuth quadruple therapy (PPI, bismuth subcitrate, metronidazole, and tetracycline; 10–14 days), concomitant therapy (PPI, clarithromycin, amoxicillin, and nitroimidazole; 10–14 days), and triple therapy (PPI, clarithromycin, and amoxicillin or metronidazole; 14 days) [5, 6]. Antibiotics have a direct and strong effect on all bacteria in the stomach [23]. The strong acid inhibitory effect of PPIs can sharply increase the stomach’s pH value, thereby reducing gastric acid’s effect on removing transient bacteria, which is not conducive to digestion and leads to various changes in substrate levels [24, 25]. Although the drugs themselves and Hp’s elimination have a potential effect on the gastric flora [19, 26], still a recent study indicated that Hp eradication might have a minimal impact on the gut microbiota [27]. There is an inverse association between Hp and the diversity of gastric microbiota [28]. The eradication of Hp might increase the diversity of gastric microbiota [20]. The relative abundance of other bacteria in the stomach might be restored after eradication to levels similar to individuals without Hp infection [20].

Using next-generation sequencing, the main phyla detected in the stomach are *Proteobacteria*, *Firmicutes*, *Bacteroidetes*, *Actinobacteria*, and *Fusobacteria* [29, 30]. The most abundant phyla change after Hp infection in the stomach, and *Proteobacteria*, *Firmicutes*, and *Actinobacteria* are the most represented [31]. The gastric cancer INS-GAS mouse model showed that the non-Hp flora could promote tumors [32, 33]. In addition, microbial diversity changes with the health of the gastric mucosal epithelium [34, 35]. Niche-specific microbial networks reflect the disease-specific microbiome, and disease-associated bacteria can form a cooperative network, contributing to the disease [36, 37]. In addition, previous research has mainly focused on the gastric microbiome of patients with gastric cancer rather than precancerous lesions such as gastritis atrophy (AG) [14, 29, 32, 34, 38, 39].

This study was developed on the hypothesis that Hp leads to change in gastric microbiota, that Hp eradication might restore gastric microbiome, and that non-Hp species might be involved in gastric lesion. Therefore, in this study, we analyzed the influence of eradication treatment on gastric flora and evaluated patients’ recovery with successful eradication under different mucosal states. The microbiome was determined based on 16S rDNA sequencing, a common approach for such analysis [35, 40]. The results could indicate whether the changes in the microbiome after eradication might be related to the change in gastric cancer risk and whether failed eradication changes the microbiome.

## Methods

### Patients and samples

Gastric biopsies (n = 152) from different anatomical sites were obtained from 89 patients at The First Affiliated Hospital of Zhejiang Chinese Medical University, Hangzhou, China. This study was approved by the ethics committee of The First Affiliated Hospital of Zhejiang Chinese Medical University. All participants provided written informed consent for participation in this study.

Patients who underwent upper gastrointestinal endoscopy and were tested for Hp were included in this study, either because of symptoms of Hp infection or non-specific gastric symptoms. In addition, in China, the patients can request a gastroscopy as part of their annual physical examination, or specific insurance plans include a gastroscopy, even in the absence of symptoms.

The exclusion criteria were (1) patients who took PPIs, H2 receptor antagonists or other antacids, probiotics, mucosal protective agents, or antibiotics in the recent 4 weeks (all these drugs have a direct effect on the gastric mucosa flora; PPIs, H2 receptor antagonists, and other antacids increase the gastric pH; probiotics are bacteria and obviously have a direct effect on the flora; mucosal protectant works by changing the mucosa of bacterial colonization; antibiotics have a direct bactericidal effect), (2) history of gastric adenoma, gastric cancer, or mucosa-associated lymphoid tissue lymphoma (all showed changes in the physiological structure of the gastric mucosa), (3) patients who underwent gastrectomy (most patients who have undergone gastrectomy have a history of advanced gastric cancer, and the normal physiological structure of the stomach has been altered), or (4) patients who underwent Hp eradication and were again Hp-positive.

Most patients underwent a ^13^C- or ^14^C-urea breath test and then gastroscopy 1–7 days later. During the endoscopic examination, one piece of gastric mucosa tissue was obtained from the antrum’s lesser curvature side and another piece from the lesser curvature side of the corpus. Each specimen was placed in a separate sterile, nonpyrogenic, and DNase/RNase-free cryopreservation tube made of polypropylene to withstand temperatures to − 196 °C (Corning Inc., Corning, NY, USA). Those specimens were kept at − 80 °C. Another mucosal biopsy of the gastric antrum was used for histological biopsy to assess gastric mucosa and Hp infection status. The ^13^C-urea breath test was performed by swallowing 75 mg of ^13^C-urea. Breath samples were collected just before and 30 min after ingestion. The ^13^C-urea breath test was considered positive when delta over baseline (DOB) was greater than 4.0%. Histological examination was performed using formalin-fixed paraffin-embedded blocks prepared from gastric biopsies. Sections were prepared and stained with Giemsa. Each glass slide was examined by a pathologist for the presence/absence of Hp. Hp was cultured from the gastric biopsies. Hp was isolated on agar (brain-heart infusion). The agar plates were incubated in a micro-aerobic environment (5% oxygen and 5–10% CO_2_) for 5 days at 37 °C.

Current Hp infection was defined as a positive result from one of the following three tests: (1) ^13^C-urea breath test, (2) histological examination, and (3) Hp culture. Furthermore, according to previous studies of the gastric microbiome, samples with < 1% of Hp relative abundance were excluded from the analysis to obtain higher representativeness [36, 41]. For patients with a history of eradication, we selected those with a completion time of 1 year. We combined the past and current Hp infection status to confirm the eradication. Only those whose gastric mucosa status was judged by endoscopy, further confirmed by pathological biopsy results, were classified into subgroups.

As per routine practice at our center, all Hp-infected patients were given a 14-day bismuth quadruple therapy consisting of omeprazole 20 mg, bismuth pectin 200 mg, furazolidone 100 mg, and amoxicillin 1000 mg, all twice daily. The outcome of the eradication therapy (for the patients who received it) was performed 4 weeks after eradication completion and was based on a ^13^C-urea breath test, histological examination, and Hp culture; any positive result was considered failed eradication. First, the patients were grouped as (a) group N (Hp-negative), (b) group P (Hp-positive), (c) group SE (Hp-positive and successful eradication), and (d) group FE (Hp-positive and failed eradication). Then based on the disease status, groups N and SE were divided into four subgroups: Hp-negative chronic gastritis [N-CG], Hp-negative gastritis atrophy [N-AG], successful-eradication chronic gastritis [SE-CG], and successful-eradication gastritis atrophy [SE-AG]).

### Analysis and testing process

#### Extraction of bacterial DNA

DNA from the different samples was extracted using the E.Z.N.A. ® Tissue DNA Kit (Omega, Inc., USA) according to the manufacturer’s instructions, which includes a bead-beating step for Gram-positive bacteria. Polymerase chain reaction (PCR) was used to amplify the 16S rDNA V3–V4 region, using the 341F 5′-CCTACGGGNGGCWGCAG-3′ and 805R 5′-GACTACHVGGGTATCTAATCC-3′ primers. The 5′ ends of the primers were tagged with specific barcodes per sample and sequencing universal primers. The thermocycler settings were (1) 98 °C for 30 s, (2) 32cycles of denaturation at 98 °C for 10 s, annealing at 54 °C for 30 s, and extension at 72 °C for 45 s, and (3) final extension at 72 °C for 10 min. PCR amplification was performed in a 25-µL reaction mixture containing 2.5 µL of each primer, 12.5 µL PCR Premix, 25 ng of template DNA, and PCR-grade water to adjust the volume. The PCR products were confirmed using 2% agarose gel electrophoresis followed by purification with AMPure XT beads (Beckman Coulter Genomics, Danvers, MA, USA) and quantified by Qubit (Invitrogen, USA). Next, the amplicon pools were prepared for sequencing, an Agilent 2100 Bioanalyzer (Agilent, USA), and the Library Quantification Kit for Illumina (Kapa Biosciences, Woburn, MA, USA) were used to assess the size and quantity of the amplicon library, respectively. The libraries were then sequenced on the NovaSeq PE250 platform.

#### Data processing

The samples were sequenced on an Illumina NovaSeq platform according to the manufacturer’s recommendations, provided by LC-Bio Technology Co., Ltd. (Hangzhou, China). Using unique barcodes, paired-end reads were assigned to samples and truncated by cutting off the barcode and primer sequence. FLASH was used to merge the paired-end reads. Quality filtering on the raw reads was performed under specific filtering conditions to obtain high-quality clean tags according to the fqtrim (v0.94). In order to ensure the accuracy and reliability of the results of subsequent analysis, the raw data were preprocessed to obtain the valid data for subsequent analysis. The primer sequences were removed. Each pair of paired-end reads were spliced into a longer tag. Windowed quality scanning was performed on the sequencing reads. The default scanning window was 100 bp. When the average quality value in the window was < 20, the part of the read from the beginning of the window to the end of 3 ‘was truncated. The truncated sequences with a length less than 100 bp were removed. The truncated sequences containing N (uncertain fuzzy base) > 5% were removed. The chimera sequences were removed. In this study, FLASH (Fast Length Adjustment of Short reads, V1.2.8, FLASH) was used to splice the double-ended sequences. Vsearch software (v2.3.4) was used to filter chimeric sequences. After dereplication using DADA2, the feature table and feature sequence were obtained. Next, we calculated alpha and beta diversities by random normalization to the same sequences. Feature abundance was normalized using the relative abundance of each sample, according to the SILVA (release 132) classifier. Alpha and beta diversities were calculated by QIIME2. Alpha diversity was determined by the observed species, Chao1, Shannon index, and Simpson index. Beta diversity was assessed by weighted UniFrac distance matrices and visualized by principal coordinate analysis (PCoA). A total of 62 pairs of samples (124 in total) were used to compare diversity. All diagrams were produced using the R package (v3.5.0). BLAST was used for sequence alignment, and the feature sequences were annotated with the SILVA database for each representative sequence.

#### Detection of differential taxa and prediction of metagenomic functions

A linear discriminant analysis (LDA) and effect size (LEfSe) were performed to determine the important bacterial taxa in the comparison group. The Phylogenetic Investigation of Communities by Reconstruction of Unobserved States 2 (PICRUSt2) program (https://github.com/picrust/picrust2) was used to infer the metagenome functional content based on the microbial community profiles obtained from the 16S rDNA gene sequences. Predicted functional genes were categorized using Kyoto Encyclopedia of Genes and Genome (KEGG) ontology (KO).

### Statistical analysis

Quantitative variables were analyzed using the Mann–Whitney U-test for the comparison of two groups. The LEfSe analysis and the comparisons of more than two groups were performed using the Kruskal–Wallis test and Wilcoxon rank-sum test. Predicted KO functions were analyzed in STAMP using the two-group comparison with White’s non-parametric t-test and corrected for multiple tests with Benjamini–Hochberg’s false discovery rate. All P-values were bilateral; a P-value < 0.05 was considered statistically significant.

## Results

### Characteristics of the patients

The characteristics of all patients are compiled in Table 1. Additional file 1: Table S1 presents the results of each patient.

**Table 1:** Characteristics of the patients

Characteristics	Age Years mean ± SD	Sex Female/Male
All patients (n = 89)	52.7 ± 14.1	45/44
Negative (n = 23)	53.5 ± 13.4	13/10
N-CG (n = 14)	46.6 ± 11.8	8/6
N-AG (n = 6)	64.7 ± 8.8	2/4
Positive (n = 17)	41.2 ± 12.6	9/8
Successful (n = 40)	58.2 ± 10.9	19/21
SE-CG (n = 8)	49.3 ± 9.9	7/1
SE-AG (n = 29)	62.1 ± 8.5	9/20
Failed (n = 9)	48.1 ± 17.8	4/5

N-CG: *Helicobacter pylori*-negative, chronic gastritis; N-AG: *Helicobacter pylori*-negative, atrophic gastritis; SE-CG: successful eradication, chronic gastritis; N-AG: successful eradication, atrophic gastritis

### Gastric antrum versus corpus mucosa

**Fig. 1:**
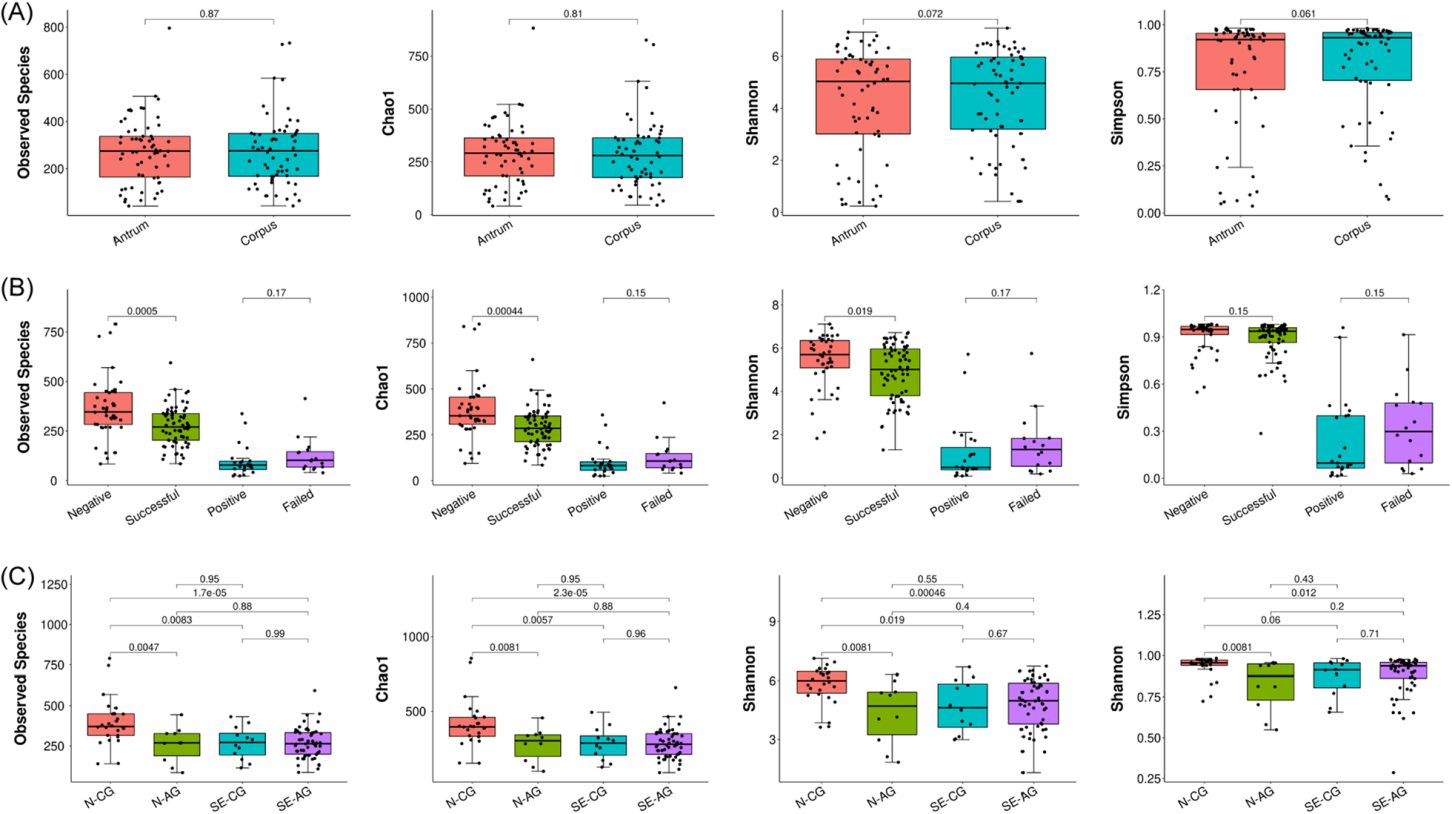
The Alpha diversity was evaluated and transformed into a box plot. The species diversity and complexity of the samples were analyzed by four indices, including Observed species, Chao1, Shannon index, and Simpson index. **A** Boxplot in the gastric antrum and corpus mucosa groups. **B** Boxplot in the four groups: Hp-Negative, Hp-Positive, Successful Eradication, and Failed Eradication. **C** Boxplot in the four subgroups: N-CG, N-AG, SE-CG, and SE-AG. Statistical significance was determined by the Wilcoxon test

**Fig. 2:**
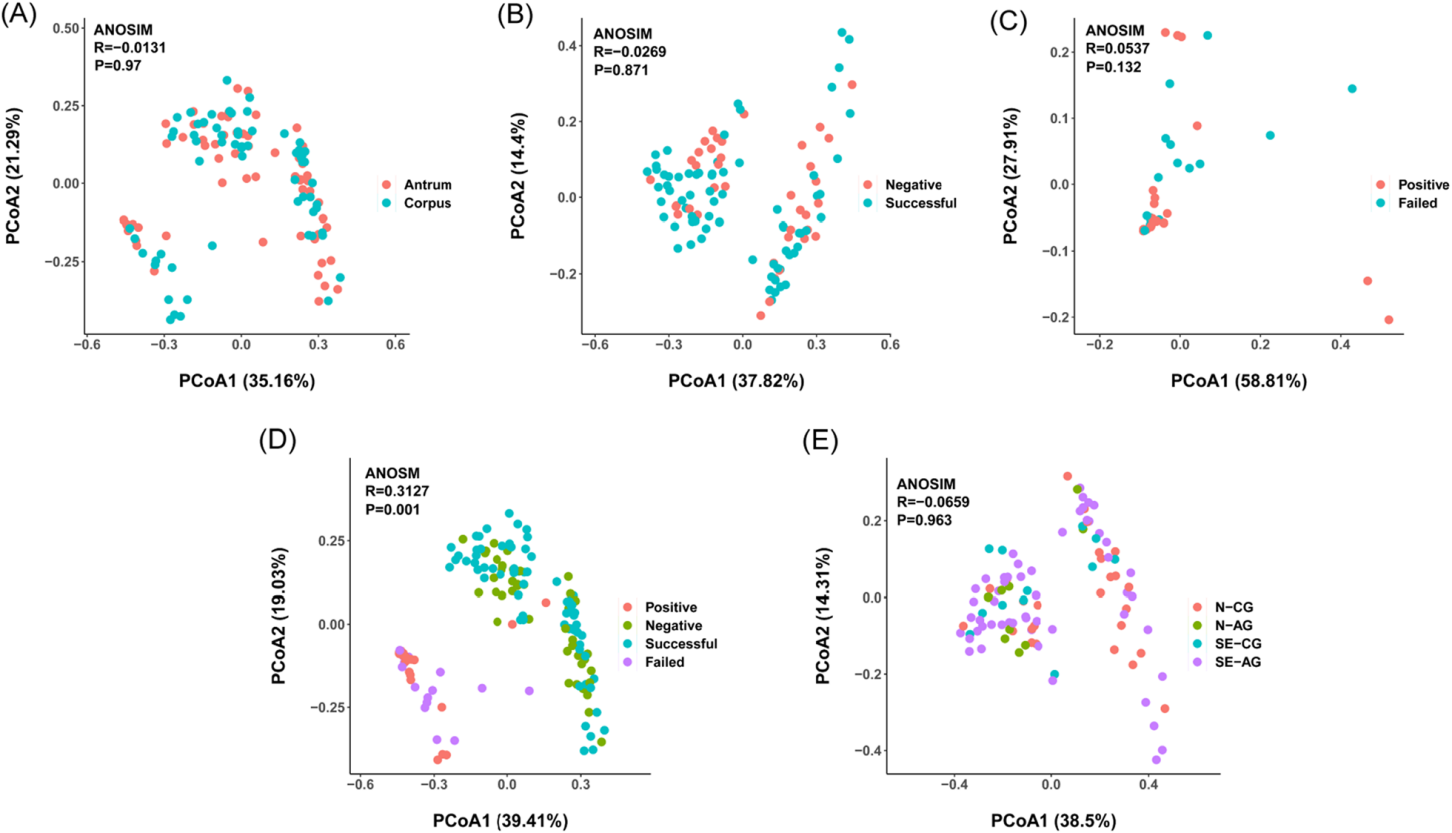
Principal coordinate analysis (PCoA) plots in which the samples were colored based on **A** paired sample location and clinical grouping: **B** Hp-Negative vs. Successful-eradication; **C** Hp-Positive vs. Failed-eradication; **D** all four groups; **E** four subgroups. ANOSIM, analysis of similarity

An average of 81,829 reads was obtained from each sample, and an average of 73,605 reads from each sample was entered into subsequent analysis after filtration. About 93.51% of the sequences were distributed on 400–500 nucleotides. An average of 8224 reads (10.05%) was filtered. The examination results of each patient are listed in Additional file 1: Table S1. In order to evaluate the alterations in the microbiota structure between the gastric antrum and corpus, we measured the microbial alpha and beta diversities. Alpha diversity showed a high degree of similarity (Fig. 1A). Beta diversity revealed no significant differences between paired sample locations (ANOSIM R = − 0.0131, P = 0.97, Fig. 2A). In Fig. 2A, the lower left included the samples from the P and FE groups, and the upper right included the samples from the N and SE groups. In other words, the lower-left group was Hp-infected, and the upper right group was Hp-uninfected. Furthermore, the LEfSe analysis (LDA > 3.5) revealed no positive results (observed species, P = 0.87; Chao1, P = 0.81; Shannon index, P = 0.072; Simpson index, P = 0.061).

### The effect of Hp on gastric flora

In the Hp-negative group, the gastric microbiota was dominated by *Firmicutes* (32.95%), *Proteobacteria* (32.26%), *Actinobacteria* (11.80%), *Bacteroidetes* (8.32%), and *Fusobacteria* (3.03%). At the same time, *Epsilonbacteraeota* (85.74%), *Proteobacteria* (4.31%), *Firmicutes* (3.21%), *Bacteroidetes* (2.72%), and *Actinobacteria* (1.49%) were the top five phyla in the Hp-positive group. According to the new classification standard, the original *Epsilonproteobacteria* (class level) is now assigned to *Epsilonbacteraeota* (phylum level), so *Helicobacter* (genus level) no longer belongs to *Proteobacteria* (phylum level) [42, 43]. Based on these changes, we described *Epsilonbacteraeota* as the dominant phylum and *Helicobacter* as the dominant genus (both over 80%) in the P and FE samples (Fig. 4A). Relative abundance of different taxa was shown as histograms (Additional file 3: Figure S1).

**Fig. 3:**
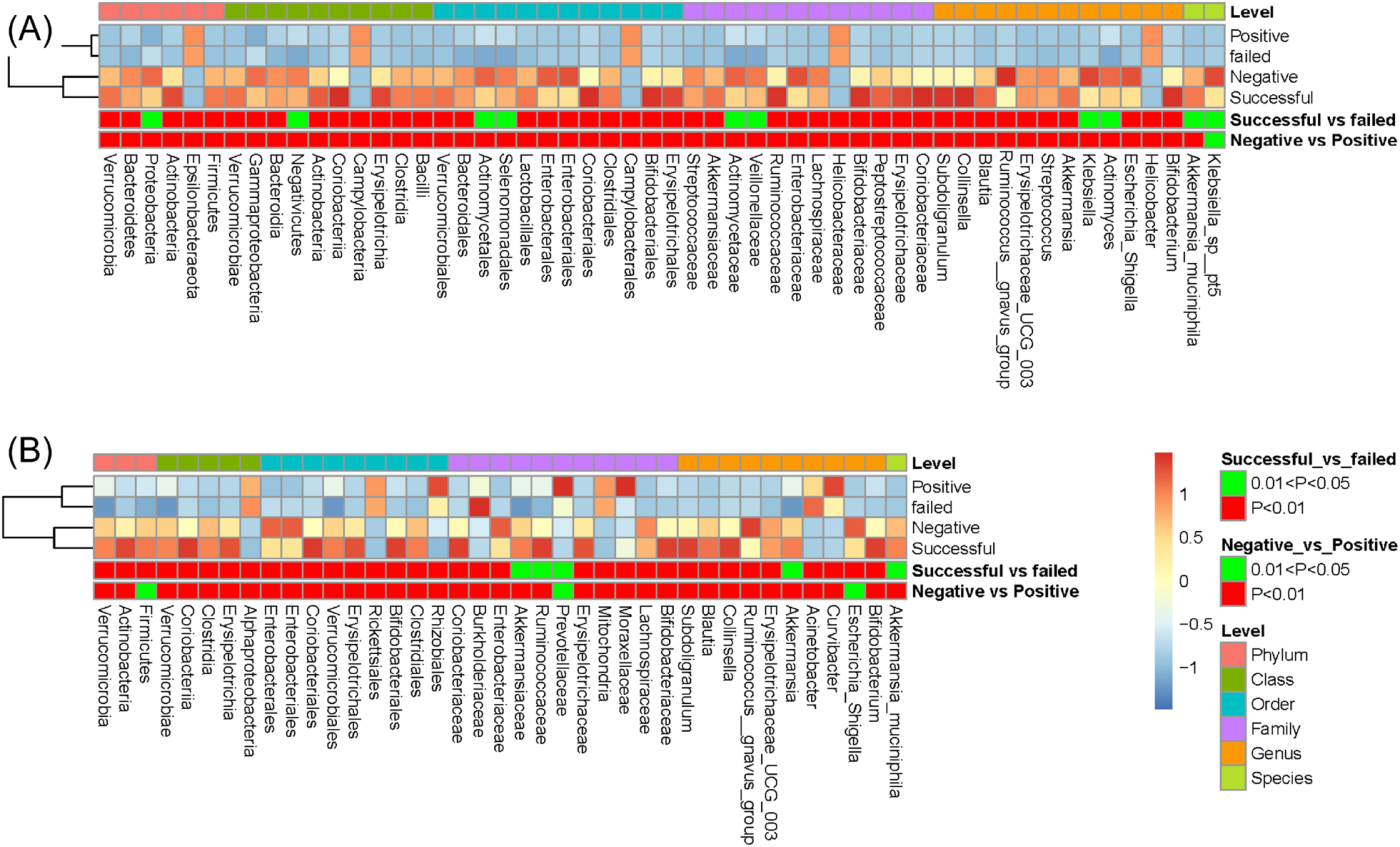
The Z-score was obtained by subtracting the average abundance and dividing it by the standard deviation of all samples. By converting the Z score into a heat map, the results of significant features (LDA score > 3.5 and adjusted P < 0.1) were displayed, including Hp (**A**) and excluding Hp (**B**) related reads, P < 0.01 and P < 0.05 are marked in red and green, respectively

In order to identify the potential biomarkers for Hp infection, we conducted a LEfSe analysis between the paired groups (groups N vs. P, groups SE vs. FE). A relative abundance cutoff was not set, as in the presence of Hp, Hp relative abundance was often very high, and the relative abundance of all other flora was low. We selected the common bacteria at different levels through the Wilcoxon rank-sum test. In groups P and FE, *Campylobacteria*, *Campylobacterales, Helicobacteraceae*, and *Helicobacter* were predominant in class, order, family, and genus levels, respectively (Fig. 3A). We performed a reanalysis by subtracting the *Helicobacter* readings from the data set to examine other taxa associated with the disease. Most genera observed a significant decrease even if the *Helicobacter* reads were removed from groups P and FE. In Fig. 3B, groups P and FE were relatively enriched for the confirmed genera *Curvibacter* and *Acinetobacter*. All potential biomarkers are shown in Fig. 3A and B. The correlation between the two genera (biomarkers) with inferred functions in the P and FE groups is provided in Additional file 3: Figure S2.

### The gastric flora after Hp eradication

**Fig. 4:**
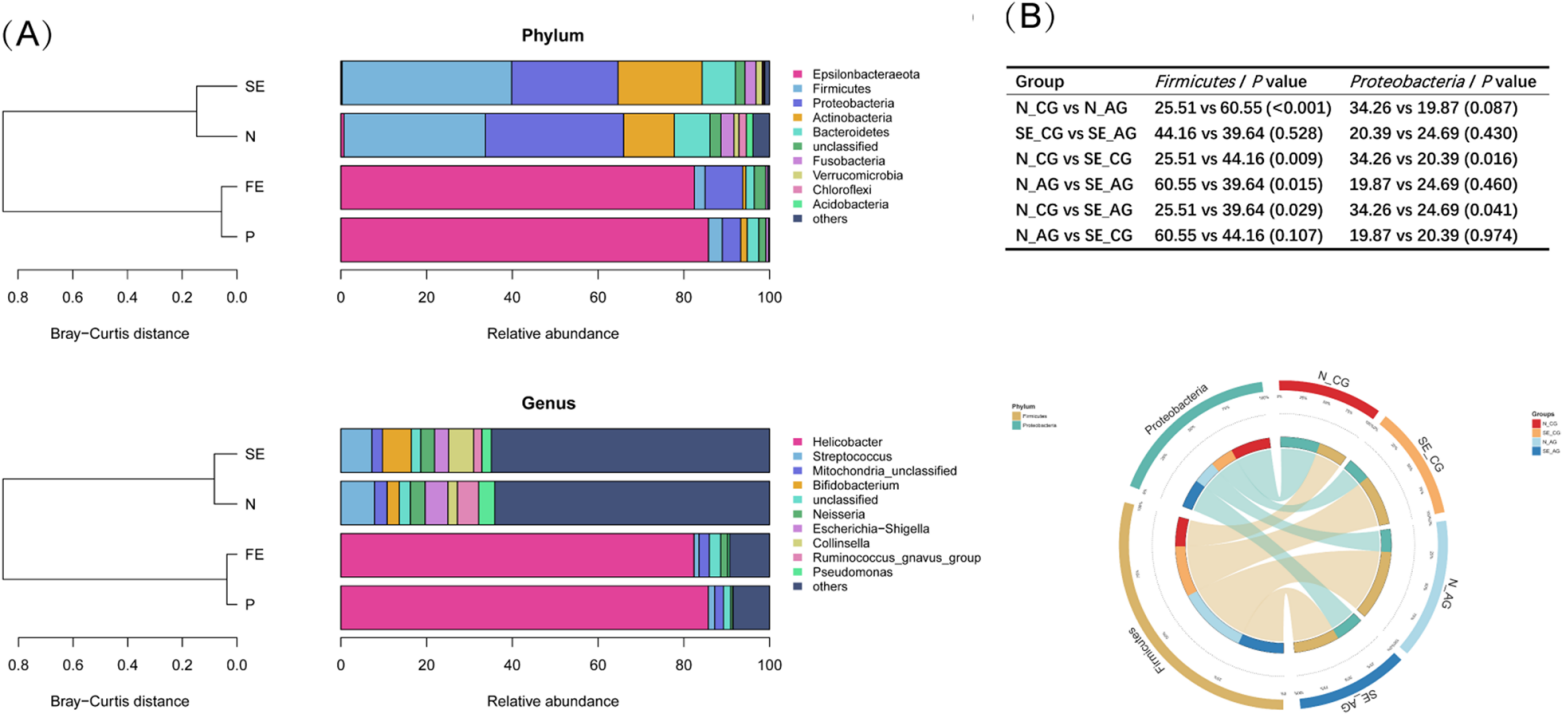
**A** Top 10 relative abundance and Bray–Curtis distance of the four groups (P, N, SE, and FE) were displayed at the phylum and genus levels. Similar bacterial composition was observed between N and SE and between P and FE. Bray–Curtis distances were used to determine the similarity of groups based on bacteria composition. **B** The average relative abundance of the two main phyla under different references in groups N and SE and subgroups were compared, and the significance was calculated by the Mann–Whitney test (P < 0.05). The average relative abundance was also shown by the Circos plot

First, we compared the gastric flora of the N and SE samples and the P and FE samples by analyzing the alpha and beta diversities. Despite the Simpson index having no significant differences in the N patients, significant reductions of the observed species, Chao1, and Shannon index were found in SE patients (Fig. 1B). No obvious differences were found between the P and FE patients (Simpson index, P = 0.15; observed species, P = 0.0005; Chao1, P = 0.00044). The beta diversity analysis revealed no remarkable differences in microbial diversity between the N and SE patients (ANOSIM R = − 0.0269, P = 0.871, Fig. 2B), as well as between the P and FE patients (ANOSIM R = 0.0537, P = 0.132, Fig. 2C) (observed species, P = 0.17; Chao1, P = 0.15; Shannon index, P = 0.17; Simpson index, P = 0.15). Figure 2B contains all the samples in the N and SE groups. When reviewing the scatter distribution of each sample, it is found that the scatter distribution of each sample is random (not regular with the antrum, corpus and subgroup). Considering that the two groups are evenly distributed on both sides, this so-called clustering may not appear after the sample size is further increased. In Fig. 2C, groups P and FE were not clustered well, mainly because of the small number of people in the two groups and also because the relative abundance of Hp varies among the samples (which was also magnified by the small number of samples). Because Hp relative abundance greater than 1% was considered to be Hp infection, although most of the samples in the P and FE groups were more than 80%, there was some low Hp relative abundance, such as the four outliers on the right, which were all low Hp relative abundance (10.47%, 21.11%, 27.40%, and 54.65%). This difference was not considered significant because the scattered points of the samples (antra and corpora) of the two groups were all reflected in the figure. Due to the high relative abundance of Hp in the matched samples, the degree of aggregation was relatively concentrated on the left side. Still, in Fig. 4D, when all the samples from the four groups were compared together, the P and FE groups appeared to be relatively well aggregated, as were the SE and N groups. When analyzing the four groups together, we distinguished two pairs of groups from sample distribution (ANOSIM R = 0.3127, P = 0.001, Fig. 2D). As the dominant phyla, the sum of the relative abundance of *Firmicutes* and *Proteobacteria* exceeded 60% in groups N and SE, and the main genera were *Streptococcus*, *Bifidobacterium*, *Escherichia-Shigella*, *Collinsella*, *Ruminococcus gnavus group*, *Neisseria*, *Pseudomonas*, and unclassified *Mitochondria* (Fig. 4A). By comparing the bacterial composition among the four groups, the bacterial composition of groups P and FE were highly similar, as well as between groups N and SE.

**Fig. 5:**
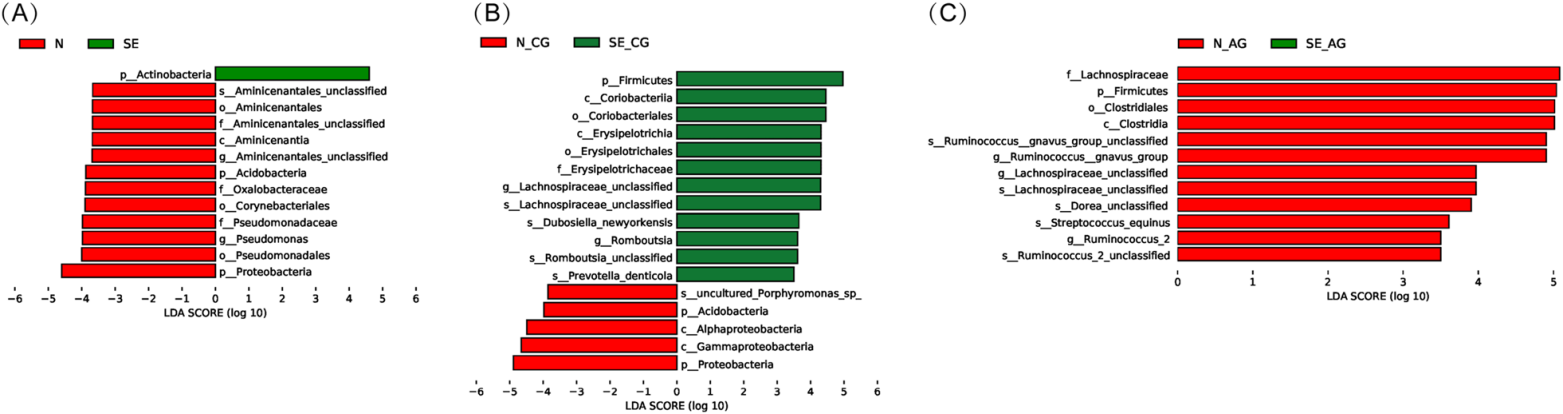
Association of specific microbiota taxa with the group of chronic gastritis and gastric carcinoma by LEfSe (LDA score > 3.5, P < 0.05). We presented the results of the analysis between N and SE (**A**), N-CG and SE-CG (**B**), and N-AG and SE-AG (**C**)

The LEfSe analysis was used to identify the potential differences in the abundance of the different bacterial taxa in groups N and SE. In the two groups, differences were found in 13 taxa (all had LDA scores > 3.5 and P < 0.05) (Fig. 5A). Relative to the N patients, SE patients exhibited preferential enrichment for *Actinobacteria*, whereas 12 bacterial taxa were preferentially depleted. Specifically, the increased genera in group N included *Pseudomonas* and unclassified *Aminicenantales*. In group FE, no taxa were distinguished from group P (Additional file 2: Table S2).

Both the SE and FE groups received eradication therapy, and the difference was whether Hp persisted or not in the gastric flora. The LEfSe analysis of the FE and P groups did not show any positive findings in bacterial flora. Figures 1B and 2D, and 4A also indicated that there were no differences in gastric bacterial flora when Hp persisted. Furthermore, in these figures, the flora of the SE group was similar to that of the N group. Overall, these results indicated that after eliminating Hp, the gastric bacterial flora could be partially restored. Lower relative abundance and richness, as mentioned in group SE, and reduced taxa implied that recovery might have some limits. No positive indicators were observed between group FE and group P, thus suggesting that the eradication treatment itself has little effect on the gastric flora.

### The different mucosal states are related to dysbacteriosis

In order to further explore the differences between CG and AG, we next analyzed the four subgroups (N-CG, N-AG, SE-CG, and SE-AG). We found that N-CG had greater richness and diversity than the other three subgroups (except for Simpson’s index comparing N-CG and SE-CG, P = 0.06), and there were no differences between the three subgroups (Fig. 1C). The sum of the relative abundance of *Firmicutes* and *Proteobacteria* exceeded 50% in each subgroup. In group N, gastric mucosal atrophy showed an increase in *Firmicutes* (P< 0.001, Fig. 4B) and was accompanied by a relative decrease in *Proteobacteria* (without statistical significance, Fig. 4B). PCoA showed that the bacterial structure was similar in all subgroups (N-CG, N-AG, SE-CG, and SE-AG, ANOSIM R = -0.0659, P = 0.963, Fig. 2E). The same trend was not observed in group SE (P = 0.528, P = 0.430, Fig. 4B). Elevated levels of *Firmicutes* taxa: genera unclassified *Lachnospiraceae* and *Romboutsia* were detected in SE-CG samples, whereas *Proteobacteria* and *Acidobacteria* taxa were depleted in these samples (Fig. 5B). Compared to N-AG, SE-AG mainly manifested as fewer *Firmicutes*, including genera *Ruminococcus gnavus group*, unclassified *Lachnospiraceae*, and *Ruminococcus 2* (Fig. 5C). Overall, gastric microbial communities were different at high taxonomic levels (e.g., genus and species compared with phylum and taxa) when comparing chronic gastritis and atrophic gastritis separately after successful eradication, indicating that the corresponding changes occurred at lower taxonomic levels (e.g., phylum and taxa compared with genus and species) as well.

### Analysis of functional changes in the gastric microbiome

**Fig. 6:**
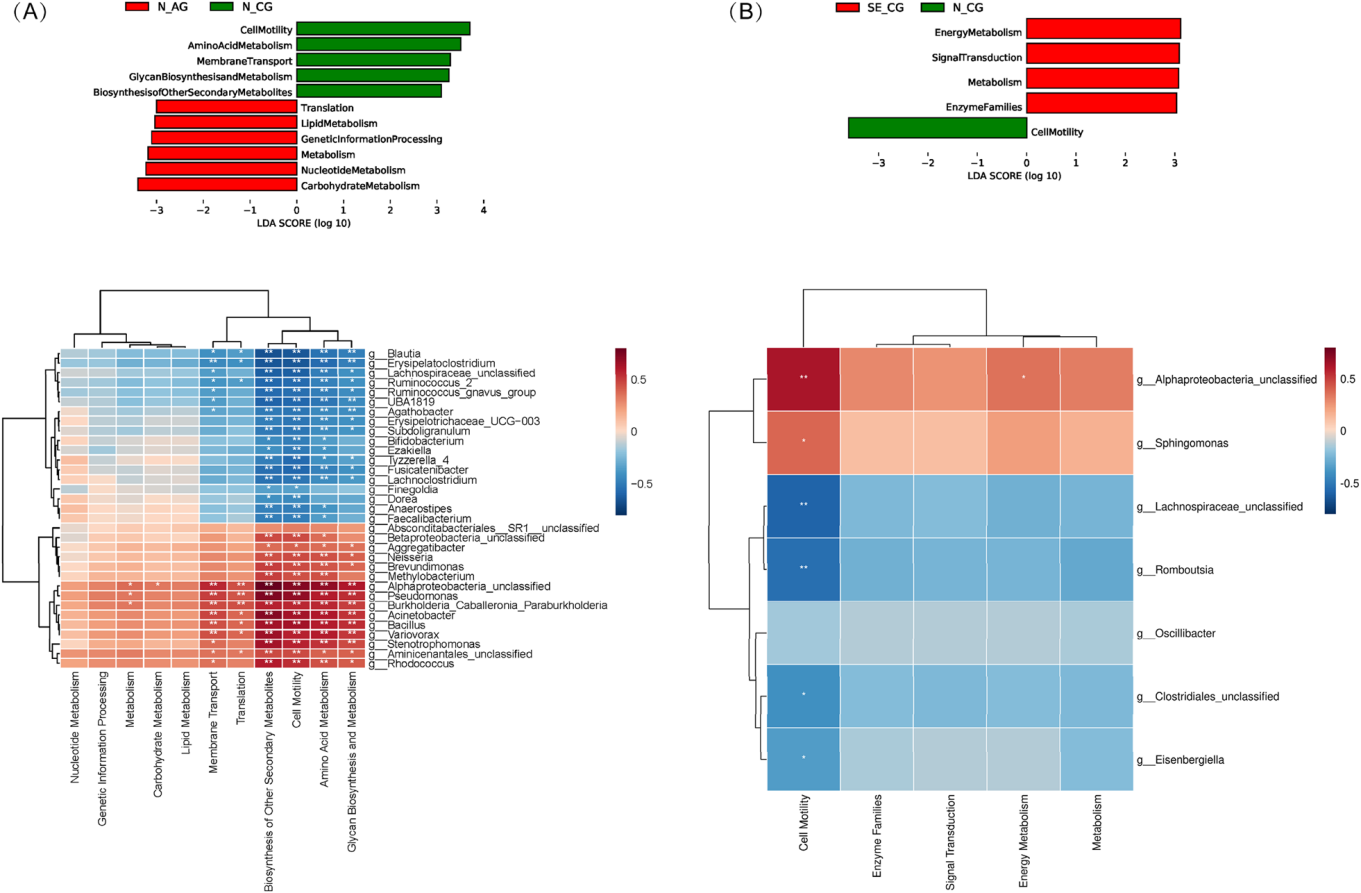
Associations of microbiota with predicted KEGG functions evaluated by Spearman correlation coefficients between 33 genera and differential KEGG pathways in N-CG versus N-AG (**A**), and between 7 genera and differential KEGG pathways in N-CG versus SE-CG (**B**). KEGG, Kyoto encyclopedia of genes and genomes

In order to infer the metagenome functional content, we used the PICRUSt2 tool based on the microbial community profiles obtained from the 16S rDNA gene sequences. Differences in putative microbiome functionality and bacterial genera between the CG and AG groups were identified using the LEfSe approach (LDA > 3). In group N, 11 identified KEGG functions were different between N-CG and N-AG (Fig. 6A), whereas no differential KEGG functions were found in subgroups (SE-CG and SE-AG) of the SE group. The subgroup SE-CG in the SE group was compared with the SE-AG subgroup in the SE group. As the results showed, the pathway involved in metabolism was overexpressed while the pathway involved in cell motility was inhibited in N-AG and SE-CG relative to N-CG (Fig. 6B). Additionally, we used correlation heatmaps to investigate the association between differential genera and KEGG pathways. Genera unclassified *Alphaproteobacteria* was positively correlated with cell motility, while genera unclassified *Lachnospiraceae* was negatively correlated with cell motility (Fig. 6A, B). Interestingly, except for four genera (*Bifidobacterium*, *Bacillus*, unclassified *Aminicenantales*, and *Rhodococcus*), all negatively correlated genera are of the phylum *Firmicutes*, while all positively correlated genera are of the phylum *Proteobacteria* (Fig. 6A, B).

The pathological changes from chronic gastritis, precancerous lesions to gastric cancer represent a long process. Other non-Hp bacteria with specific functions are likely to be involved. The existing hypothesis is that nitrate-reducing bacterial species are associated with an increased risk of gastric carcinoma [26]. Eradication might have a potential impact on this function by changing the flora in the stomach. Therefore, we evaluated four subgroups and compared the results. Pairwise comparisons revealed that all nitrate and nitrite reductase functions had no significant differences (Additional file 3: Figure S3), suggesting that these functions were still at low levels in both CG and AG stages relative to gastric cancer.

## Discussion

In the present study, we identified differential bacterial taxa and metagenomic functions before and after successful Hp eradication. Based on our results, the bacterial composition between the paired gastric antrum and corpus was highly similar, supported by previous studies [20, 44, 45].

In the 16S rDNA gene variable regions, V3-V4 shows the highest taxonomic coverage, diversity, reproducibility, and PCR-amplification efficiency [46], and the primer set was determined to have high efficiency [47, 48]. 16S full-length sequencing requires third-generation sequencing methods, and its price is relatively high. The NovaSeq is sufficient for most taxonomic applications [49]. Qubit was used to quantify the library, and the qualified library concentration had to be above 2 nM. Gradient dilution was required, and then the mixture was mixed in the corresponding proportion according to the required sequencing volume. After NaOH denaturation, the Reagent was NovaSeq 6000 SP Reagent Kit (500 cycles), with at least 50,000 tags per sample. The HiSeq platform enables PE250 sequencing, which can obtain the same length of reads as MiSeq, but the volume and quality of sequencing data are much higher than MiSeq. When using the PE250 sequencing mode, HiSeq generates 10 times more data than MiSeq, allowing thousands of samples to be tested simultaneously. Compared with the HiSeq platform, the NovaSeq platform has higher data volume output, faster sequencing speed, higher sequencing quality, and more comprehensive application scenarios. It is more suitable for microbiome experiments such as amplification sequencing and metagenomic sequencing with a large sample size and data volume [50, 51].

Hp has the greatest impact on gastric bacterial composition and diversity [20]. In this study, we found that the abundance and diversity of bacteria after Hp colonization were significantly lower than in non-infected individuals. In addition, regardless of Hp infection, *Proteobacteria* was reported as the dominant bacterial group in the stomach [20, 52]. Still, as the latest research no longer classifies Hp within *Proteobacteria*, *Epsilonbacteraeota* has become the most common phylum in Hp-infected patients [42, 43]. Despite this, the identified genera *Curvibacter* and *Acinetobacter* associated with Hp infection still belong to *Proteobacteria*. *Curvibacter*, which is a common part of the oral microflora, is prevalent in patients with atherosclerotic plaques [53], but the role of *Curvibacter* in gastric lesions remains to be explored. Earlier studies reported that atrophic gastritis was accompanied by a reduction in Hp colonization [38, 54]. Ofori-Darko et al. concluded that the OmpA-like protein from *Acinetobacter* spp. could stimulate gastrin and IL-8 cytokine production, which suggests they can cause gastritis or participate in the transformation towards atrophic gastritis [55]. *Acinetobacter* appeared to be enriched after Hp colonization (Fig. 3B compared to the relatively low level in the SE and N groups without Hp colonization), and thus might play an important role in Hp pathogenesis. The LEfSe analysis showed that *Acinetobacter* was enriched in N-CG group compared with N-AG group. As shown in Fig. 6A, cell motility, amino acid metabolism, glycan biosynthesis and metabolism, membrane transport and biosynthesis of other secondary metabolites were positively correlated with *Acinetobacter* in N-CG. In addition to Hp itself, *Acinetobacter* might also be involved in the development of atrophic gastritis. Therefore, it was reasonable to speculate that this non-Hp bacteria might not only participate in the development of atrophic gastritis when Hp is present but also play a role alone when Hp colonization is reduced or eradicated.

The richness, diversity, and structure of the bacterial communities in the FE and P samples were highly similar. The results showed that once Hp occupies the stomach, it is difficult to disturb the gastric flora. Hence, Hp in the FE group remained dominant even after the eradication treatment, and its flora composition did not change. In the case of failure of Hp eradication, the effect of eradication drugs on the gastric flora appears to be limited. After successful eradication of Hp, the phylum and genus composition of the gastric flora could be restored to levels close to those of Hp-negative subjects, and the bacterial diversity index increased, which was consistent with previous reports [20, 35]. Still, there was a significant difference between the SE and N groups, revealing an outcome of limited recovery. We assumed that these differences might be due to some irreversible changes after Hp colonization. In order to confirm this, we evaluated whether gastric mucosal atrophy could affect the intragastric flora through four subgroups (N-CG, N-AG, SE-CG, SE-AG). The results showed that the richness and diversity of Hp-negative CG patients were significantly higher than those of the other subgroups, and no significant differences were observed among them (N-AG, SE-CG, and SE-AG). As an initiating factor, Hp is crucial in the progression of gastric mucosa from chronic gastritis to atrophic gastritis or even gastric cancer [12, 26, 56]. Still, Hp might not be the only causative factor in the process. This further confirmed our hypothesis that regardless of the gastric mucosal atrophy development, the gastric flora of patients with successful eradication was closer to N-AG. Hp often spontaneously disappears in elderly patients because of the progression of atrophic gastritis [57], which was not examined in this study but will have to be studied in the future as it could support the role of non-Hp species in the development of gastric cancer. Thus, while Hp infection might initiate atrophic gastritis, it might not mediate the final transforming events [58]. In addition, other bacterial species might initiate these events [59]. The bacterial driver could explain it, i.e., the passenger model in which Hp initiates the long carcinogenic process but is not a persisting factor during the process [60]. Some irreversible changes may occur after Hp colonization. Moreover, the proportion of *Proteobacteria* and *Firmicutes* was similar in the SE-CG and SE-AG groups. Thus, we speculated that Hp colonization probably accelerates the changes in the flora of SE-CG patients to atrophied flora. From this perspective, patients with successful eradication still seem to be at a higher risk than the normal population.

Recent studies showed that in the gastric carcinoma microbiota, increased nitrate reductase and nitrite reductase functions were considered as drivers of cancer development [37, 39, 61]. In order to assess this risk, we next addressed the functional features of the microbiota. Still, our results did not reveal this trend in atrophy patients. We speculate that the risk of dysbacteriosis in this regard is relatively low due to the successful elimination of Hp.

To sum up, this article focused on the impact of eradication on the flora and assessment of the recovery of patients with successful Hp eradication. Moreover, we described the effects of gastric mucosal atrophy on the changes in gastric microbiota. Our study verified that in the presence of Hp, the gastric flora was quite stable and, therefore, difficult to alter by antibiotics and highly effective acid suppressants. Hp is the initiating factor and a key link in Correa’s cascade [26]. Moreover, even when advanced precancerous lesions occur, the successful removal of Hp is of great importance, especially in East Asia [62]. Thus, it seems that the risk of gastric cancer in patients with successful eradication has been greatly reduced, which has been confirmed by large-scale clinical research [21]. Still, for those who already experienced precancerous lesions such as atrophy, the risk is higher than in the normal aging stomach [22]. Consistent with this, our study reported that people who successfully eradicated Hp were closer to those with Hp-negative gastric mucosal atrophy, which represented a smaller bacterial community. Interestingly, this change might not have a profound impact. Still, a previous study showed that *Peptostreptococcus*, *Streptococcus*, *Parvimonas*, *Prevotella*, *Rothia*, and *Granulicatella* were associated with emergence and persistence of gastric atrophy and intestinal metaplasia 1 year after eradication [63]. Future studies should examine the microbiome over time, from before to after eradication and during follow-up, in relation to the development of lesions.

This study has a few limitations. First, this was a single-center cross-sectional study with a small sample size, especially considering those enrolled in groups P and FE. Still, in this study, we implemented strict screening criteria and excluded the samples with < 1% of Hp sequence to obtain higher representativeness. Secondly, we did not obtain mucosal samples from the same subjects before and after Hp eradication treatment to achieve self-control. Third, the bacterial community is continuous and dynamic, so it was impossible to determine the causal relationship between these changes and different states. In addition, this study did not use PCR quantification techniques to quantify individual bacteria in different samples; thus, the analysis could only be based on the relative abundance of different bacteria. Finally, regarding causality, this study had a cross-sectional design, and causality could not be determined. It was added as a limitation. Therefore, further studies are still needed to verify and clarify the influence of eradication and precancerous lesions on gastric microbiome.

## Conclusions

After Hp infection, the diversity and relative abundance of gastric microflora were significantly decreased. Yet, gastric microbiota could be partially restored to the Hp-negative status after successful eradication. Still, this effect was incomplete and might contribute to the long-term risks. The specific mechanisms and pathways underlying these changes will be explored in future research.

## Supplementary Information

**Additional file 1: Table S1.** The examination results of each patient.

**Additional file 2: Table S2.** The LEfSe analysis of the FE and P groups.

**Additional file 3: Figure S1.** The histograms for the top10 bacterium in different taxa. **Figure S2.** Associations of biomarkers with inferred functions. **Figure S3.** Prediction of nitrate reductase and nitrite reductase functions.

## Data Availability

Data are available upon reasonable request from the authors.
