# Supplementary material for: Impact of Helicobacter pylori eradication on the gastric microbiome
Source: Gut Pathog. 2021 Oct 13;13:60. doi: 10.1186/s13099-021-00460-2 (PMC8513236; doi:10.1186/s13099-021-00460-2)
Supplement: Supplementary file 3 — Additional file 3: Figure S1. The histograms for the top10 bacterium in different taxa. Figure S2. Associations of biomarkers with inferred functions. Figure S3. Prediction of nitrate reductase and nitrite reductase functions. [file 13099_2021_460_MOESM3_ESM.pdf]

Supplementary figures

Figure S1. Relative abundance of Phyla (A), Classes (B), Orders (C), Families (D), Genera (E) and Species (F) in each group of patients. P, Hp-Positive; FE, Fail eradication; N, Hp-Negative; SE, Successful eradication.

Figure S2. Associations of different taxa after *Helicobacter* reads were removed. The heatmaps show partial Spearman correlation coefficients between P and FE groups (A), between N and SE groups (B). P, Hp-positive; FE, Fail eradication; N, Hp-negative; SE, Successful eradication.

Figure S3. The normalised relative frequency of nitrate reductase and nitrite reductase in patients with chronic gastritis and gastric carcinoma is shown. (A) N-CG versus SE-CG. (B) N-AG versus SE-AG. (C) N-CG versus N-AG. (D) SE-CG versus SE-AG. Significance was considered for adjusted $P<0.05$. N-CG, Hp-negative chronic gastritis; N-AG, Hp-negative atrophic gastritis; SE-CG, successful-eradication chronic gastritis; SE-AG, successful-eradication atrophic gastritis; NADH, nicotinamide adenine dinucleotide; NO, nitric oxide.

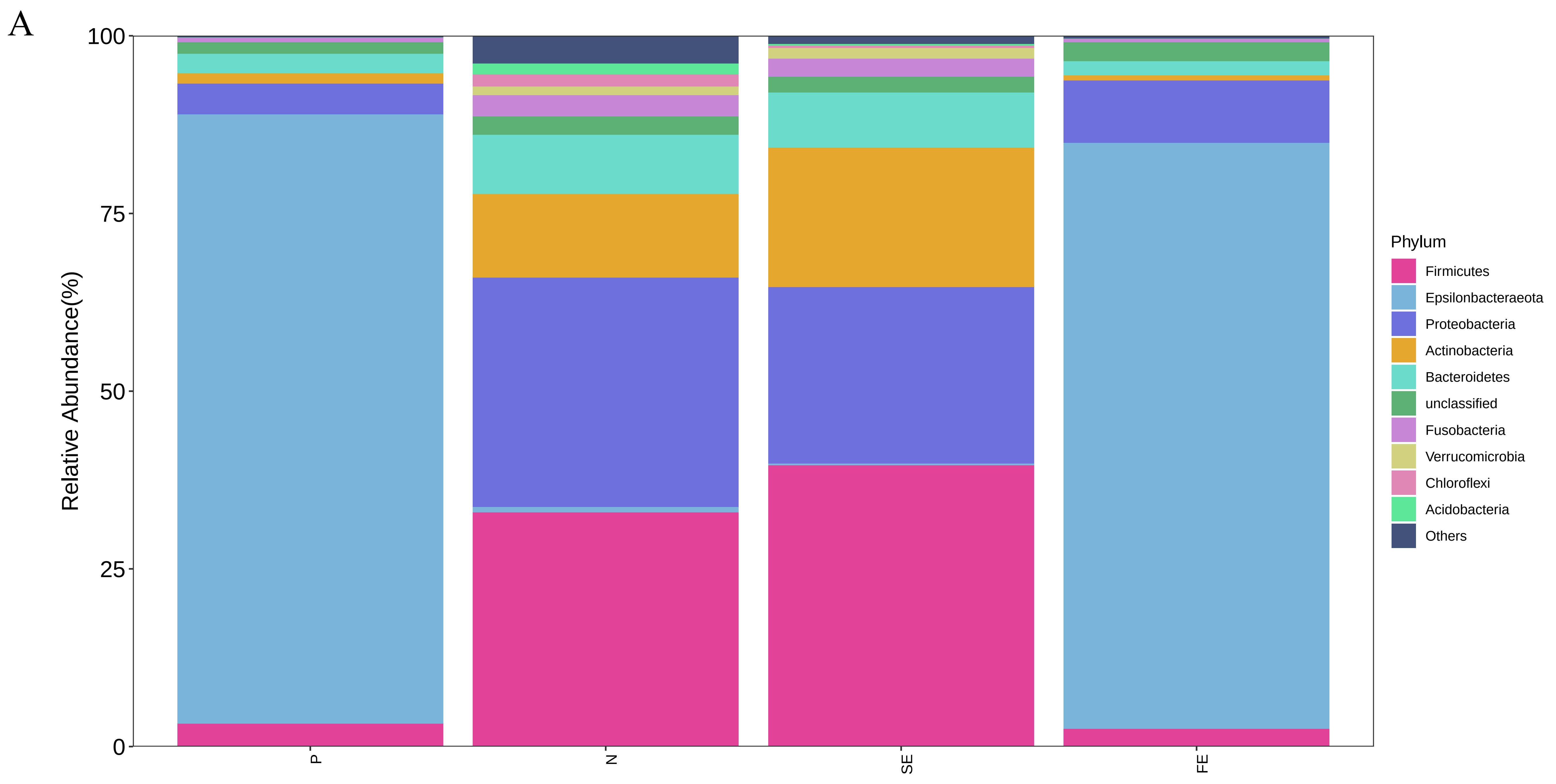

A
100
75
50
25
0
Relative Abundance(%)
P
N
SE
FE
Phylum
Firmicutes
Epsilonbacteraeota
Proteobacteria
Actinobacteria
Bacteroidetes
unclassified
Fusobacteria
Verrucomicrobia
Chloroflexi
Acidobacteria
Others

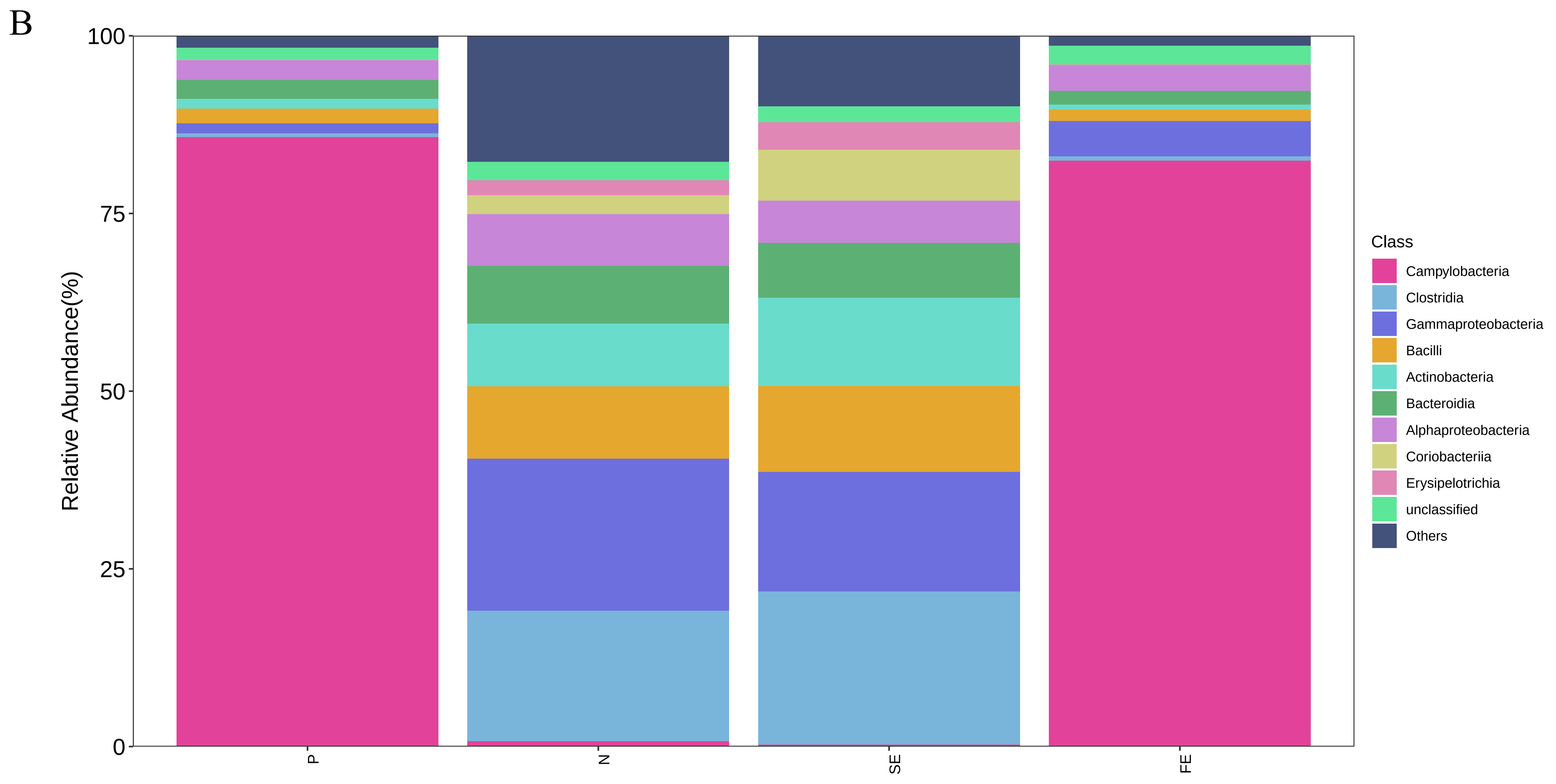
B
Relative Abundance(%)
100
75
50
25
0
P
N
SE
FE
Class
Campylobacteria
Clostridia
Gammaproteobacteria
Bacilli
Actinobacteria
Bacteroidia
Alphaproteobacteria
Coriobacteriia
Erysipelotrichia
unclassified
Others

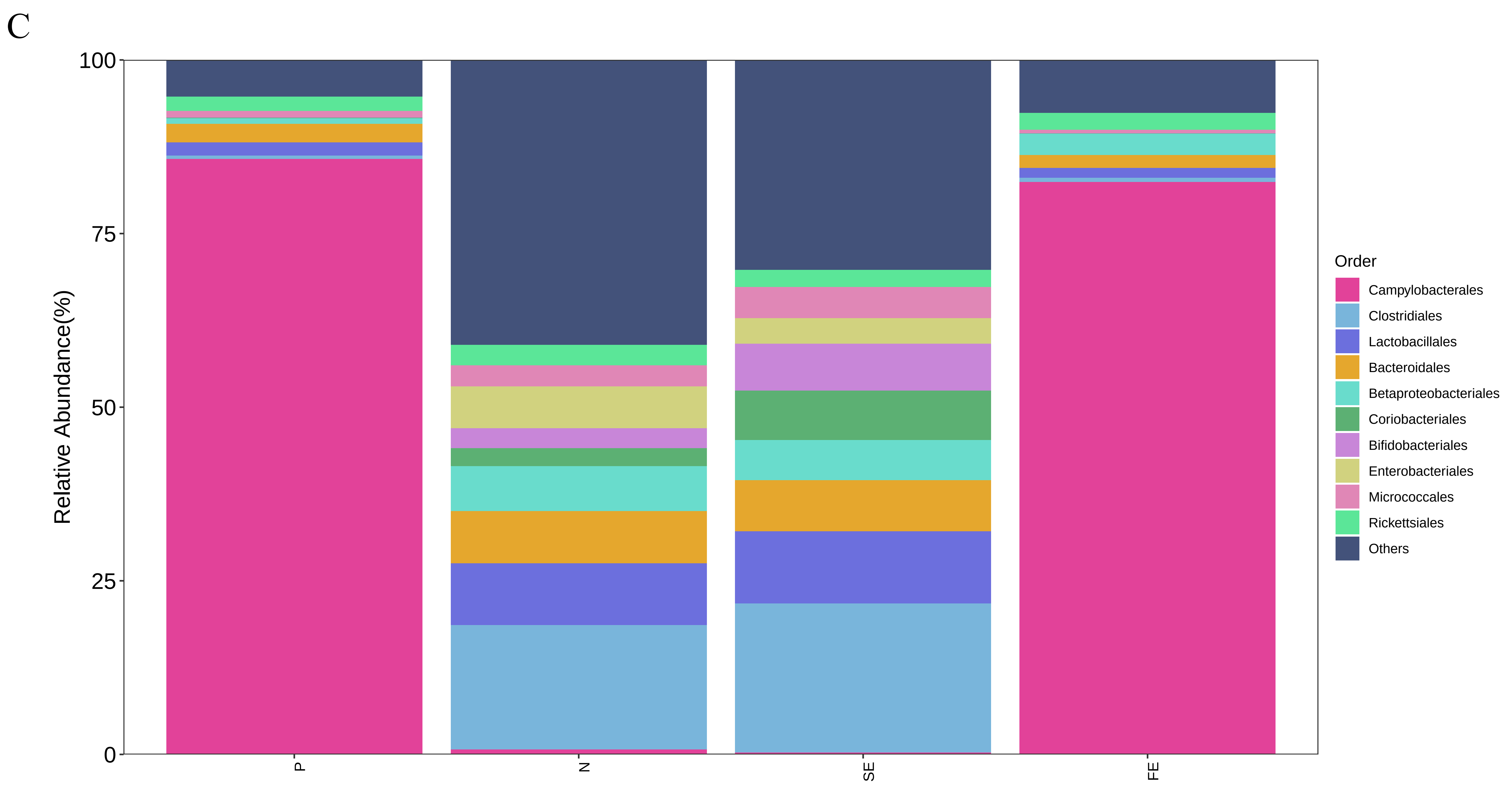
C
100
75
50
25
0
Relative Abundance(%)
P
N
SE
FE
Order
Campylobacterales
Clostridiales
Lactobacillales
Bacteroidales
Betaproteobacteriales
Coriobacteriales
Bifidobacteriales
Enterobacteriales
Micrococcales
Rickettsiales
Others

D

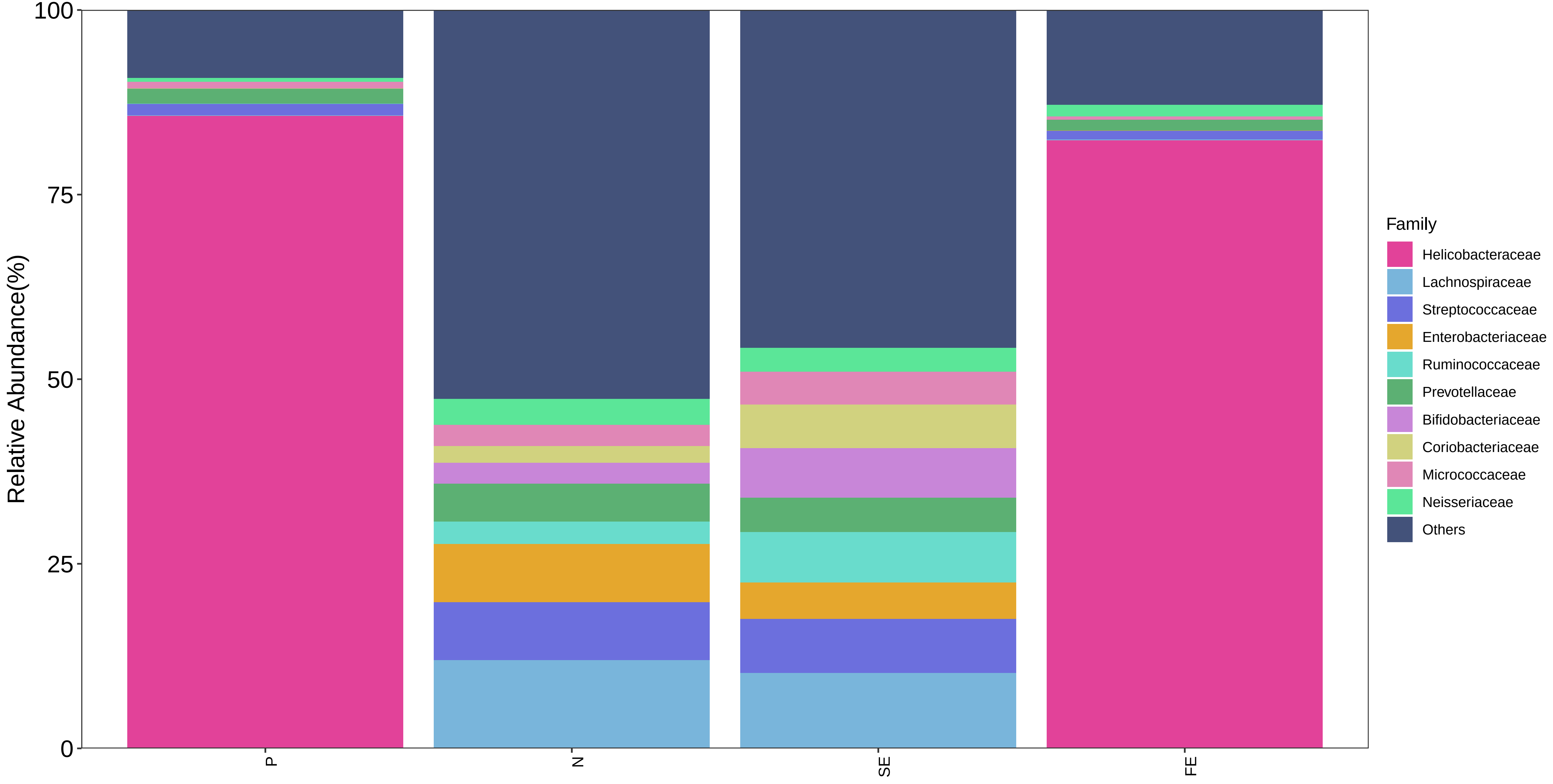

100
75
50
25
0
Relative Abundance(%)
P
N
SE
FE
Family
Helicobacteraceae
Lachnospiraceae
Streptococcaceae
Enterobacteriaceae
Ruminococcaceae
Prevotellaceae
Bifidobacteriaceae
Coriobacteriaceae
Micrococcaceae
Neisseriaceae
Others

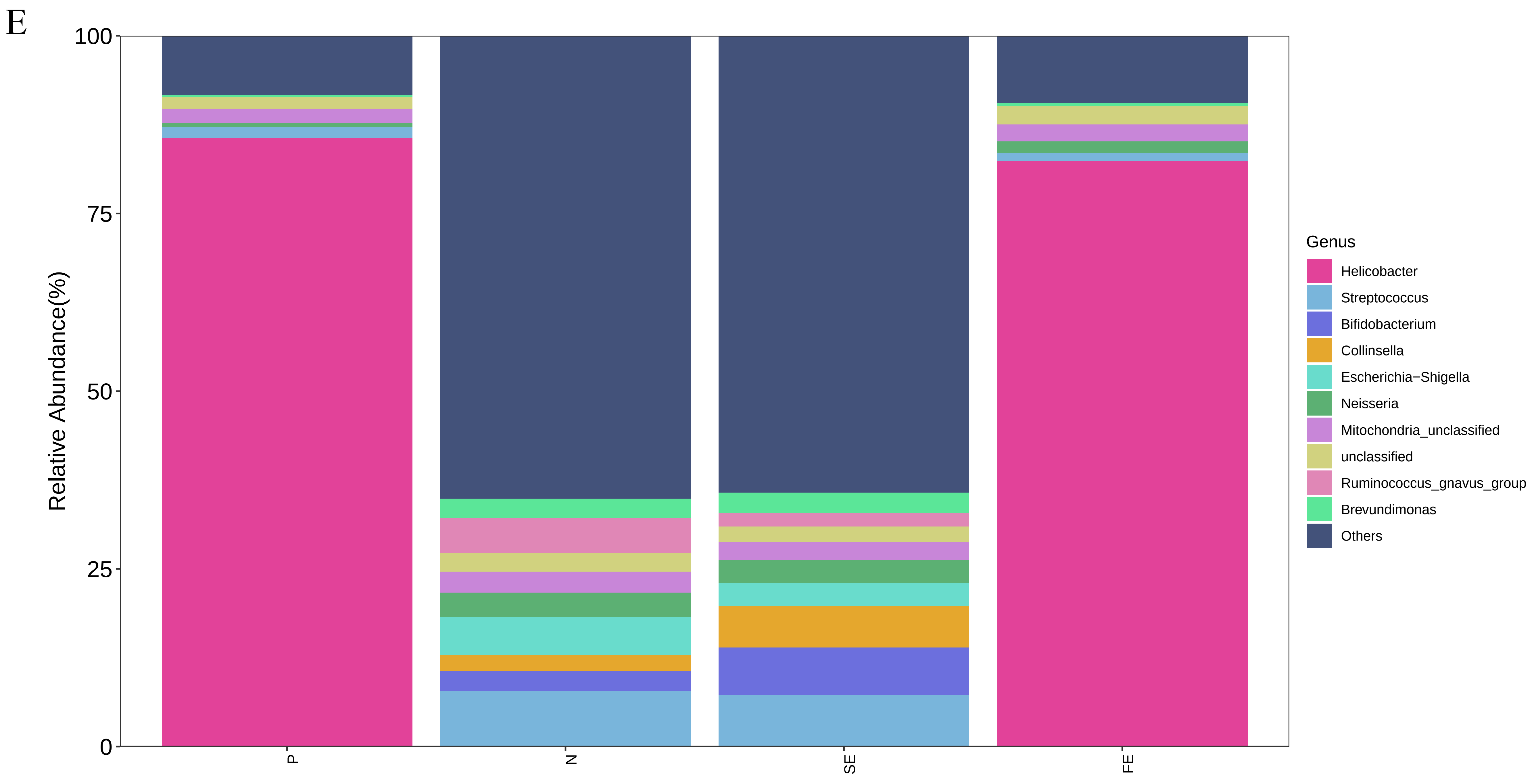
E
Relative Abundance(%)
100
75
50
25
0
P
N
SE
FE
Genus
Helicobacter
Streptococcus
Bifidobacterium
Collinsella
Escherichia−Shigella
Neisseria
Mitochondria_unclassified
unclassified
Ruminococcus_gnavus_group
Brevundimonas
Others

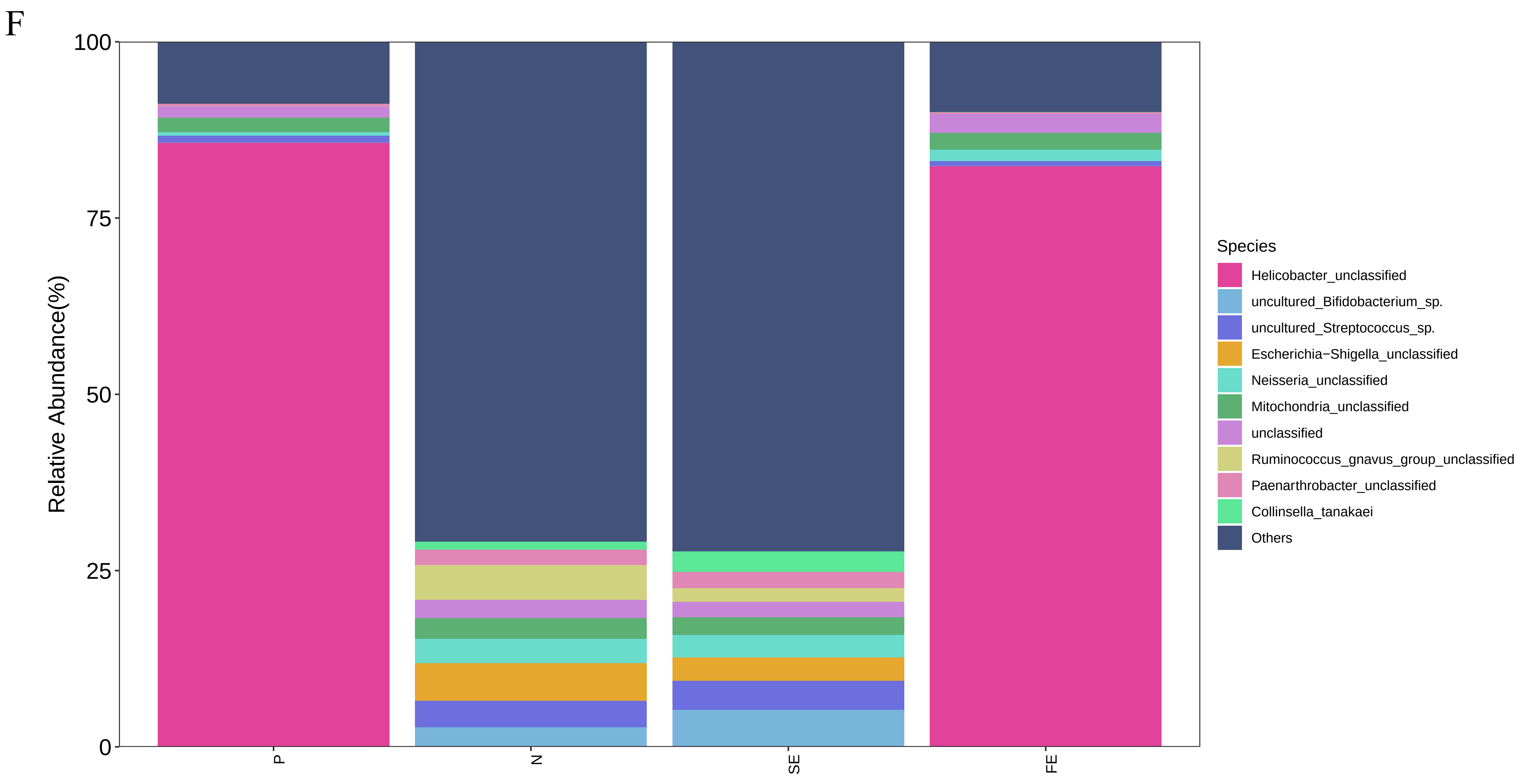

F
100
75
50
25
0
Relative Abundance(%)
P
N
SE
FE
Species
Helicobacter_unclassified
uncultured_Bifidobacterium_sp.
uncultured_Streptococcus_sp.
Escherichia−Shigella_unclassified
Neisseria_unclassified
Mitochondria_unclassified
unclassified
Ruminococcus_gnavus_group_unclassified
Paenarthrobacter_unclassified
Collinsella_tanakaei
Others

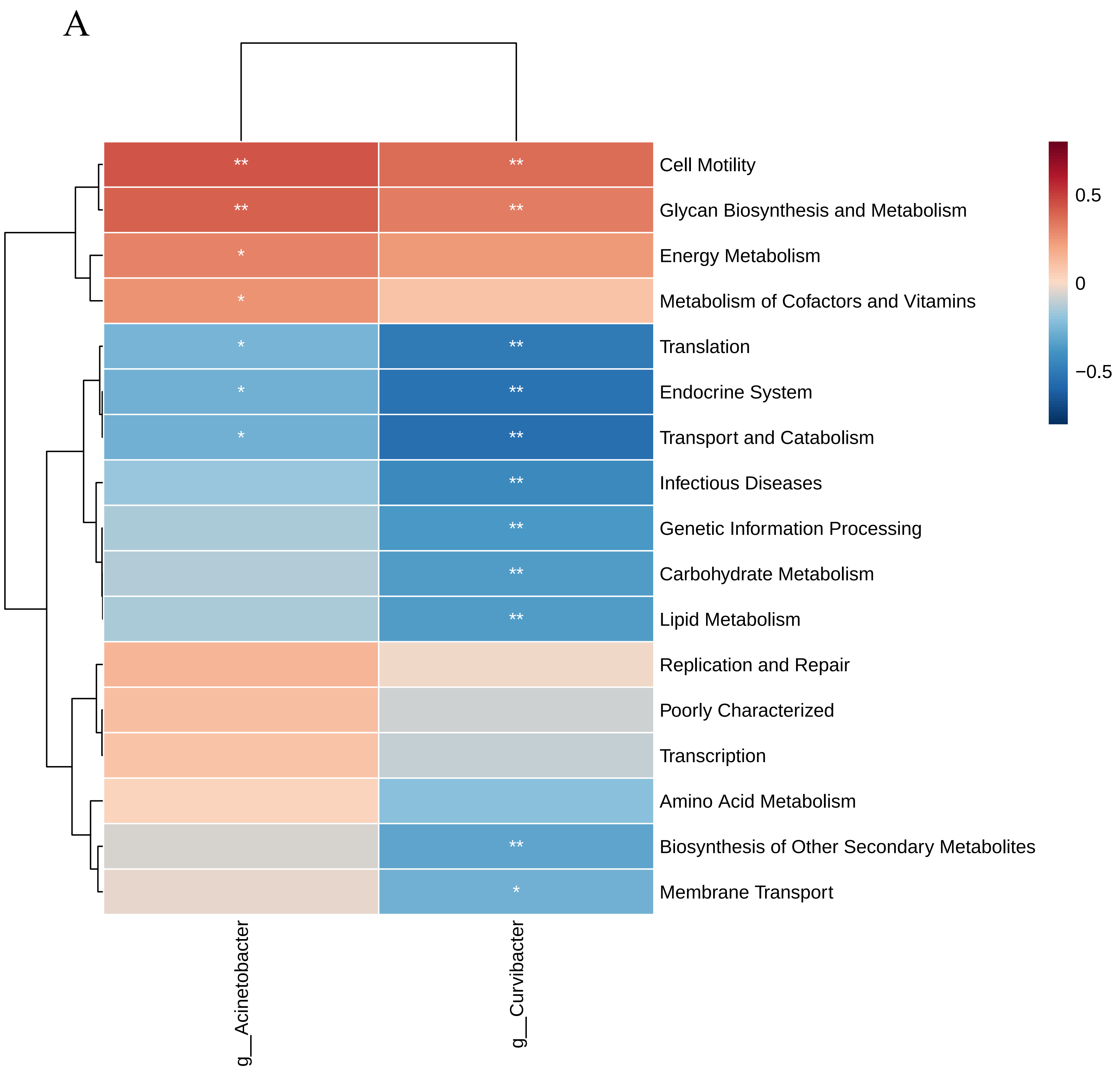
A
Cell Motility
Glycan Biosynthesis and Metabolism
Energy Metabolism
Metabolism of Cofactors and Vitamins
Translation
Endocrine System
Transport and Catabolism
Infectious Diseases
Genetic Information Processing
Carbohydrate Metabolism
Lipid Metabolism
Replication and Repair
Poorly Characterized
Transcription
Amino Acid Metabolism
Biosynthesis of Other Secondary Metabolites
Membrane Transport
g__Acinetobacter
g__Curvibacter
0.5
0
−0.5

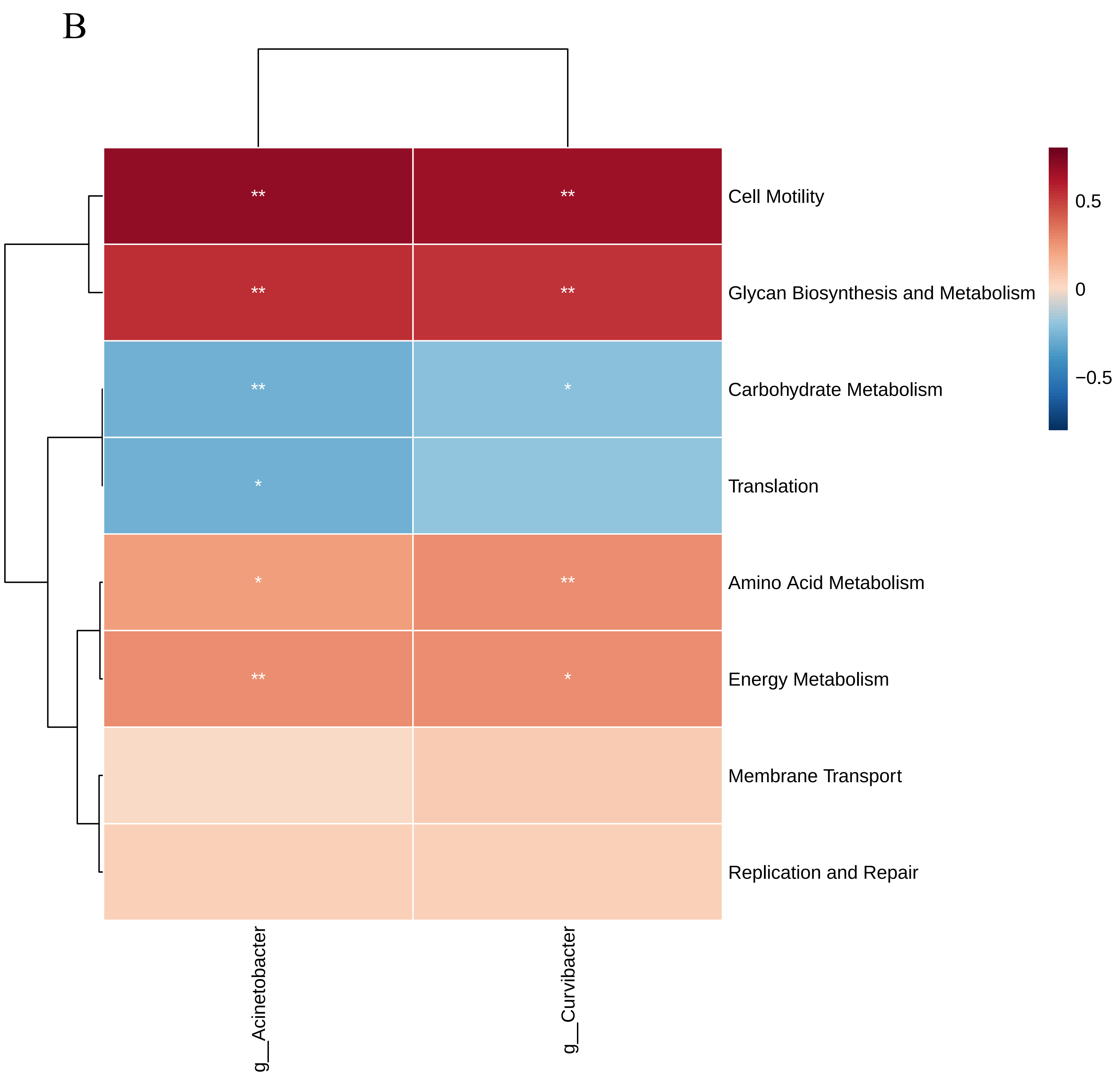
B
Cell Motility
Glycan Biosynthesis and Metabolism
Carbohydrate Metabolism
Translation
Amino Acid Metabolism
Energy Metabolism
Membrane Transport
Replication and Repair
g__Acinetobacter
g__Curvibacter
0.5
0
−0.5

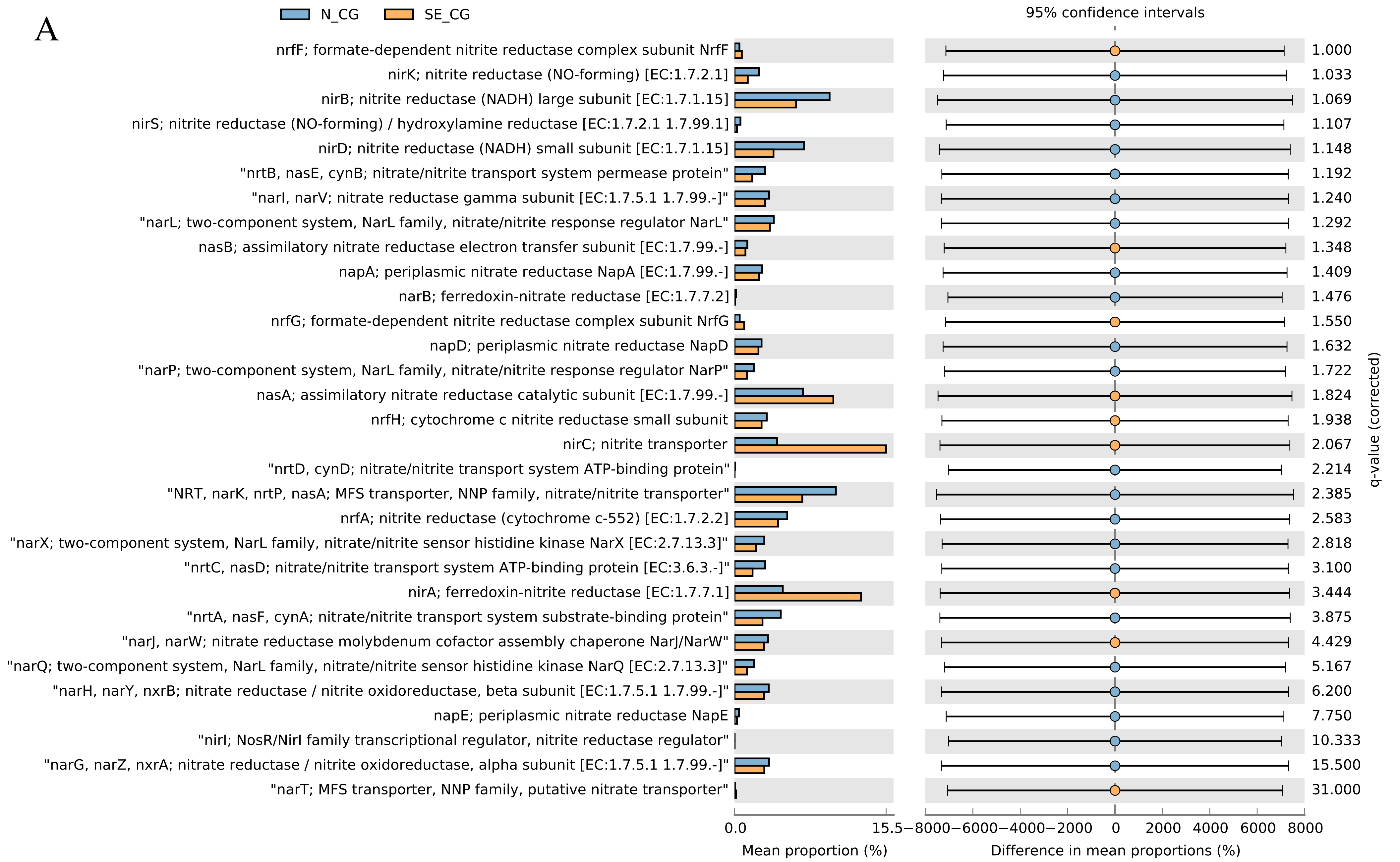

A
N_CG
SE_CG
95% confidence intervals
nrfF; formate-dependent nitrite reductase complex subunit NrfF
nirK; nitrite reductase (NO-forming) [EC:1.7.2.1]
nirB; nitrite reductase (NADH) large subunit [EC:1.7.1.15]
nirS; nitrite reductase (NO-forming) / hydroxylamine reductase [EC:1.7.2.1 1.7.99.1]
nirD; nitrite reductase (NADH) small subunit [EC:1.7.1.15]
"nrtB, nasE, cynB; nitrate/nitrite transport system permease protein"
"narI, narV; nitrate reductase gamma subunit [EC:1.7.5.1 1.7.99.-]"
"narL; two-component system, NarL family, nitrate/nitrite response regulator NarL"
nasB; assimilatory nitrate reductase electron transfer subunit [EC:1.7.99.-]
napA; periplasmic nitrate reductase NapA [EC:1.7.99.-]
narB; ferredoxin-nitrate reductase [EC:1.7.7.2]
nrfG; formate-dependent nitrite reductase complex subunit NrfG
napD; periplasmic nitrate reductase NapD
"narP; two-component system, NarL family, nitrate/nitrite response regulator NarP"
nasA; assimilatory nitrate reductase catalytic subunit [EC:1.7.99.-]
nrfH; cytochrome c nitrite reductase small subunit
nirC; nitrite transporter
"nrtD, cynD; nitrate/nitrite transport system ATP-binding protein"
"NRT, narK, nrtP, nasA; MFS transporter, NNP family, nitrate/nitrite transporter"
nrfA; nitrite reductase (cytochrome c-552) [EC:1.7.2.2]
"narX; two-component system, NarL family, nitrate/nitrite sensor histidine kinase NarX [EC:2.7.13.3]"
"nrtC, nasD; nitrate/nitrite transport system ATP-binding protein [EC:3.6.3.-]"
nirA; ferredoxin-nitrite reductase [EC:1.7.7.1]
"nrtA, nasF, cynA; nitrate/nitrite transport system substrate-binding protein"
"narJ, narW; nitrate reductase molybdenum cofactor assembly chaperone NarJ/NarW"
"narQ; two-component system, NarL family, nitrate/nitrite sensor histidine kinase NarQ [EC:2.7.13.3]"
"narH, narY, nxrB; nitrate reductase / nitrite oxidoreductase, beta subunit [EC:1.7.5.1 1.7.99.-]"
napE; periplasmic nitrate reductase NapE
"nirI; NosR/NirI family transcriptional regulator, nitrite reductase regulator"
"narG, narZ, nxrA; nitrate reductase / nitrite oxidoreductase, alpha subunit [EC:1.7.5.1 1.7.99.-]"
"narT; MFS transporter, NNP family, putative nitrate transporter"
1.000
1.033
1.069
1.107
1.148
1.192
1.240
1.292
1.348
1.409
1.476
1.550
1.632
1.722
1.824
1.938
2.067
2.214
2.385
2.583
2.818
3.100
3.444
3.875
4.429
5.167
6.200
7.750
10.333
15.500
31.000
q-value (corrected)
0.0
15.5
−8000
−6000
−4000
−2000
0
2000
4000
6000
8000
Mean proportion (%)
Difference in mean proportions (%)

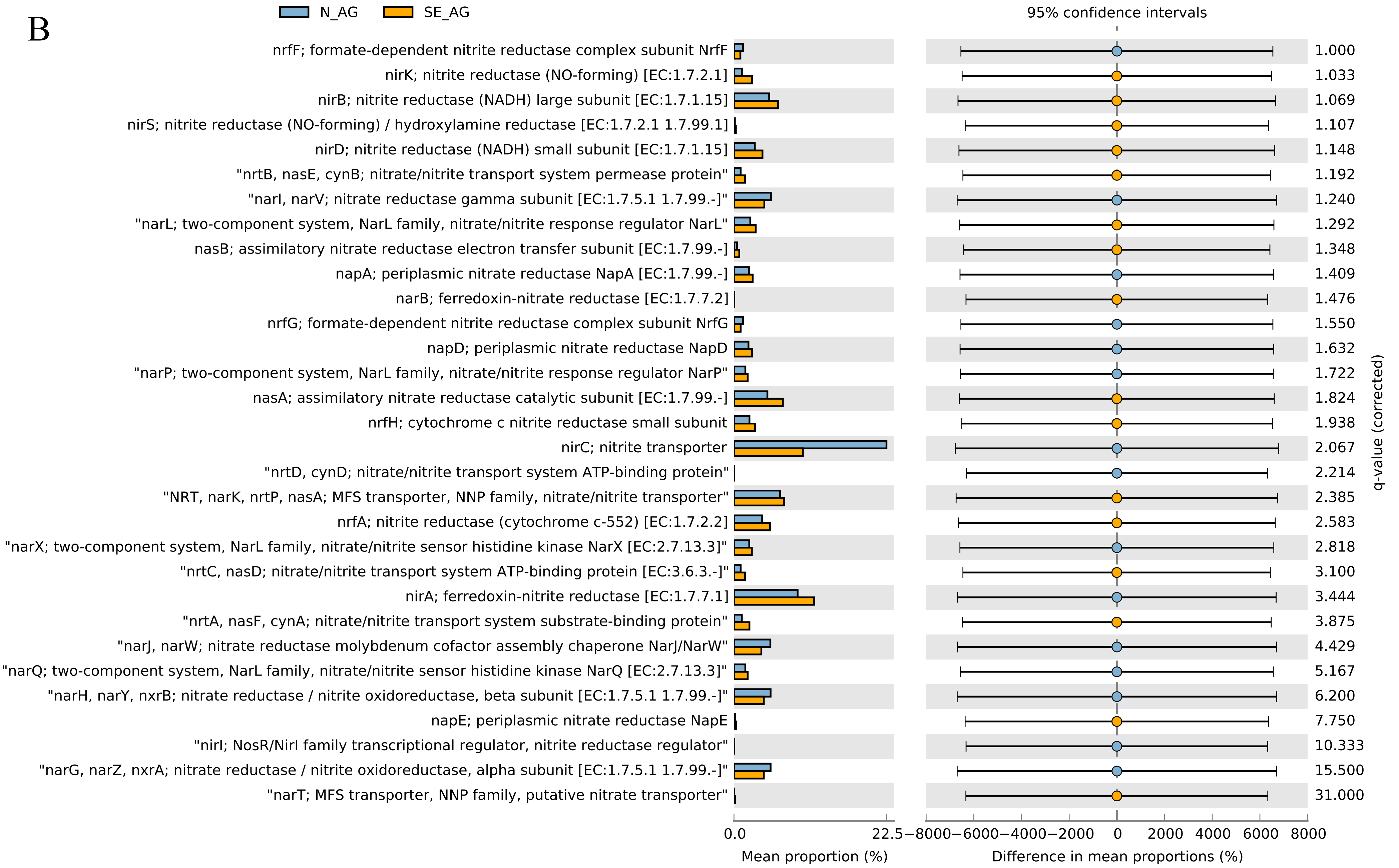

B
N_AG
SE_AG
95% confidence intervals
nrfF; formate-dependent nitrite reductase complex subunit NrfF
nirK; nitrite reductase (NO-forming) [EC:1.7.2.1]
nirB; nitrite reductase (NADH) large subunit [EC:1.7.1.15]
nirS; nitrite reductase (NO-forming) / hydroxylamine reductase [EC:1.7.2.1 1.7.99.1]
nirD; nitrite reductase (NADH) small subunit [EC:1.7.1.15]
"nrtB, nasE, cynB; nitrate/nitrite transport system permease protein"
"narI, narV; nitrate reductase gamma subunit [EC:1.7.5.1 1.7.99.-]"
"narL; two-component system, NarL family, nitrate/nitrite response regulator NarL"
nasB; assimilatory nitrate reductase electron transfer subunit [EC:1.7.99.-]
napA; periplasmic nitrate reductase NapA [EC:1.7.99.-]
narB; ferredoxin-nitrate reductase [EC:1.7.7.2]
nrfG; formate-dependent nitrite reductase complex subunit NrfG
napD; periplasmic nitrate reductase NapD
"narP; two-component system, NarL family, nitrate/nitrite response regulator NarP"
nasA; assimilatory nitrate reductase catalytic subunit [EC:1.7.99.-]
nrfH; cytochrome c nitrite reductase small subunit
nirC; nitrite transporter
"nrtD, cynD; nitrate/nitrite transport system ATP-binding protein"
"NRT, narK, nrtP, nasA; MFS transporter, NNP family, nitrate/nitrite transporter"
nrfA; nitrite reductase (cytochrome c-552) [EC:1.7.2.2]
"narX; two-component system, NarL family, nitrate/nitrite sensor histidine kinase NarX [EC:2.7.13.3]"
"nrtC, nasD; nitrate/nitrite transport system ATP-binding protein [EC:3.6.3.-]"
nirA; ferredoxin-nitrite reductase [EC:1.7.7.1]
"nrtA, nasF, cynA; nitrate/nitrite transport system substrate-binding protein"
"narJ, narW; nitrate reductase molybdenum cofactor assembly chaperone NarJ/NarW"
"narQ; two-component system, NarL family, nitrate/nitrite sensor histidine kinase NarQ [EC:2.7.13.3]"
"narH, narY, nxrB; nitrate reductase / nitrite oxidoreductase, beta subunit [EC:1.7.5.1 1.7.99.-]"
napE; periplasmic nitrate reductase NapE
"nirI; NosR/NirI family transcriptional regulator, nitrite reductase regulator"
"narG, narZ, nxrA; nitrate reductase / nitrite oxidoreductase, alpha subunit [EC:1.7.5.1 1.7.99.-]"
"narT; MFS transporter, NNP family, putative nitrate transporter"
1.000
1.033
1.069
1.107
1.148
1.192
1.240
1.292
1.348
1.409
1.476
1.550
1.632
1.722
1.824
1.938
2.067
2.214
2.385
2.583
2.818
3.100
3.444
3.875
4.429
5.167
6.200
7.750
10.333
15.500
31.000
q-value (corrected)
0.0
22.5
Mean proportion (%)
−8000
−6000
−4000
−2000
0
2000
4000
6000
8000
Difference in mean proportions (%)

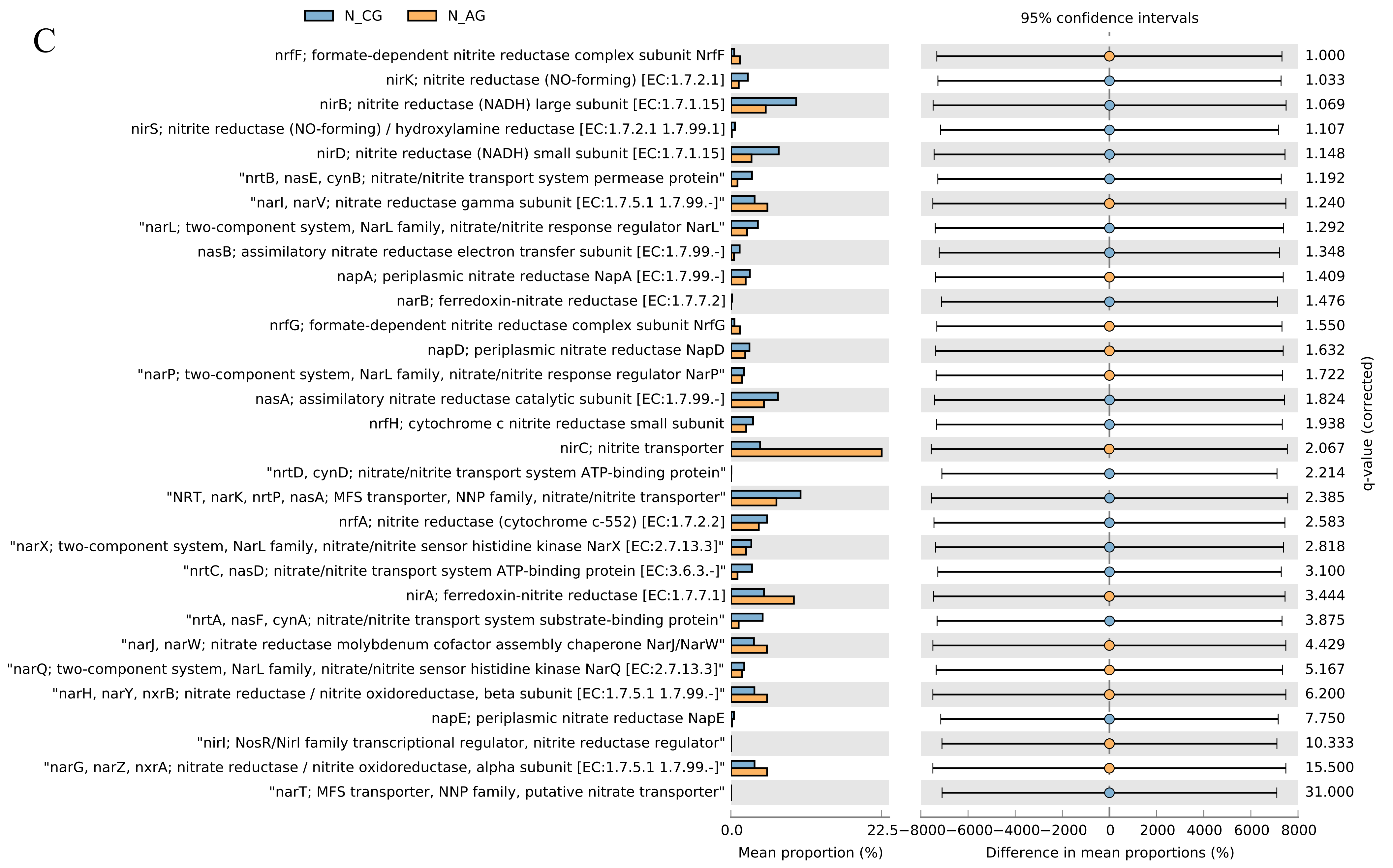

C
N_CG
N_AG
95% confidence intervals
nrfF; formate-dependent nitrite reductase complex subunit NrfF
nirK; nitrite reductase (NO-forming) [EC:1.7.2.1]
nirB; nitrite reductase (NADH) large subunit [EC:1.7.1.15]
nirS; nitrite reductase (NO-forming) / hydroxylamine reductase [EC:1.7.2.1 1.7.99.1]
nirD; nitrite reductase (NADH) small subunit [EC:1.7.1.15]
"nrtB, nasE, cynB; nitrate/nitrite transport system permease protein"
"narI, narV; nitrate reductase gamma subunit [EC:1.7.5.1 1.7.99.-]"
"narL; two-component system, NarL family, nitrate/nitrite response regulator NarL"
nasB; assimilatory nitrate reductase electron transfer subunit [EC:1.7.99.-]
napA; periplasmic nitrate reductase NapA [EC:1.7.99.-]
narB; ferredoxin-nitrate reductase [EC:1.7.7.2]
nrfG; formate-dependent nitrite reductase complex subunit NrfG
napD; periplasmic nitrate reductase NapD
"narP; two-component system, NarL family, nitrate/nitrite response regulator NarP"
nasA; assimilatory nitrate reductase catalytic subunit [EC:1.7.99.-]
nrfH; cytochrome c nitrite reductase small subunit
nirC; nitrite transporter
"nrtD, cynD; nitrate/nitrite transport system ATP-binding protein"
"NRT, narK, nrtP, nasA; MFS transporter, NNP family, nitrate/nitrite transporter"
nrfA; nitrite reductase (cytochrome c-552) [EC:1.7.2.2]
"narX; two-component system, NarL family, nitrate/nitrite sensor histidine kinase NarX [EC:2.7.13.3]"
"nrtC, nasD; nitrate/nitrite transport system ATP-binding protein [EC:3.6.3.-]"
nirA; ferredoxin-nitrite reductase [EC:1.7.7.1]
"nrtA, nasF, cynA; nitrate/nitrite transport system substrate-binding protein"
"narJ, narW; nitrate reductase molybdenum cofactor assembly chaperone NarJ/NarW"
"narQ; two-component system, NarL family, nitrate/nitrite sensor histidine kinase NarQ [EC:2.7.13.3]"
"narH, narY, nxrB; nitrate reductase / nitrite oxidoreductase, beta subunit [EC:1.7.5.1 1.7.99.-]"
napE; periplasmic nitrate reductase NapE
"nirI; NosR/NirI family transcriptional regulator, nitrite reductase regulator"
"narG, narZ, nxrA; nitrate reductase / nitrite oxidoreductase, alpha subunit [EC:1.7.5.1 1.7.99.-]"
"narT; MFS transporter, NNP family, putative nitrate transporter"
1.000
1.033
1.069
1.107
1.148
1.192
1.240
1.292
1.348
1.409
1.476
1.550
1.632
1.722
1.824
1.938
2.067
2.214
2.385
2.583
2.818
3.100
3.444
3.875
4.429
5.167
6.200
7.750
10.333
15.500
31.000
q-value (corrected)
0.0
22.5
−8000
−6000
−4000
−2000
0
2000
4000
6000
8000
Mean proportion (%)
Difference in mean proportions (%)

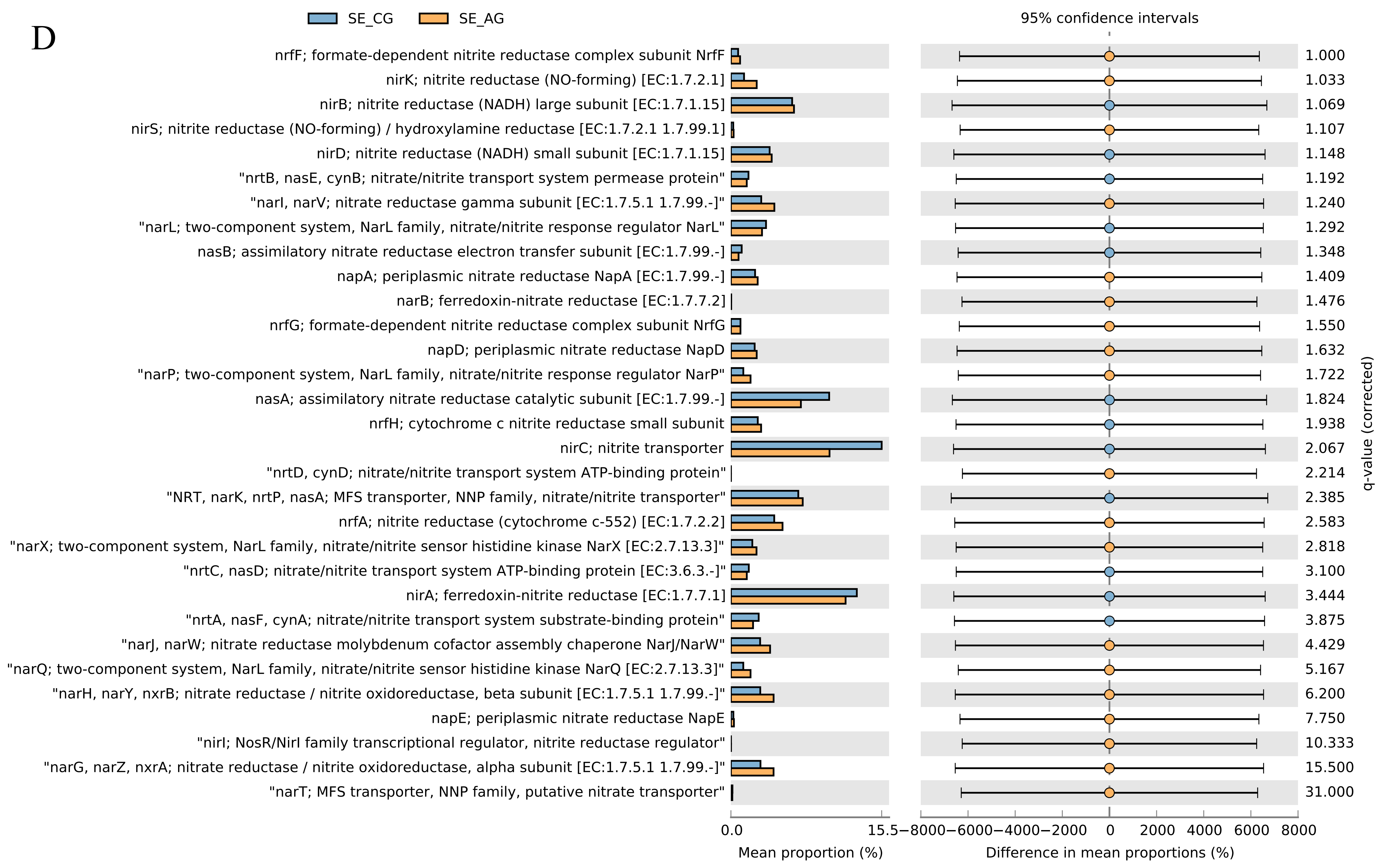

D
SE_CG
SE_AG
95% confidence intervals
nrfF; formate-dependent nitrite reductase complex subunit NrfF
nirK; nitrite reductase (NO-forming) [EC:1.7.2.1]
nirB; nitrite reductase (NADH) large subunit [EC:1.7.1.15]
nirS; nitrite reductase (NO-forming) / hydroxylamine reductase [EC:1.7.2.1 1.7.99.1]
nirD; nitrite reductase (NADH) small subunit [EC:1.7.1.15]
"nrtB, nasE, cynB; nitrate/nitrite transport system permease protein"
"narI, narV; nitrate reductase gamma subunit [EC:1.7.5.1 1.7.99.-]"
"narL; two-component system, NarL family, nitrate/nitrite response regulator NarL"
nasB; assimilatory nitrate reductase electron transfer subunit [EC:1.7.99.-]
napA; periplasmic nitrate reductase NapA [EC:1.7.99.-]
narB; ferredoxin-nitrate reductase [EC:1.7.7.2]
nrfG; formate-dependent nitrite reductase complex subunit NrfG
napD; periplasmic nitrate reductase NapD
"narP; two-component system, NarL family, nitrate/nitrite response regulator NarP"
nasA; assimilatory nitrate reductase catalytic subunit [EC:1.7.99.-]
nrfH; cytochrome c nitrite reductase small subunit
nirC; nitrite transporter
"nrtD, cynD; nitrate/nitrite transport system ATP-binding protein"
"NRT, narK, nrtP, nasA; MFS transporter, NNP family, nitrate/nitrite transporter"
nrfA; nitrite reductase (cytochrome c-552) [EC:1.7.2.2]
"narX; two-component system, NarL family, nitrate/nitrite sensor histidine kinase NarX [EC:2.7.13.3]"
"nrtC, nasD; nitrate/nitrite transport system ATP-binding protein [EC:3.6.3.-]"
nirA; ferredoxin-nitrite reductase [EC:1.7.7.1]
"nrtA, nasF, cynA; nitrate/nitrite transport system substrate-binding protein"
"narJ, narW; nitrate reductase molybdenum cofactor assembly chaperone NarJ/NarW"
"narQ; two-component system, NarL family, nitrate/nitrite sensor histidine kinase NarQ [EC:2.7.13.3]"
"narH, narY, nxrB; nitrate reductase / nitrite oxidoreductase, beta subunit [EC:1.7.5.1 1.7.99.-]"
napE; periplasmic nitrate reductase NapE
"nirI; NosR/NirI family transcriptional regulator, nitrite reductase regulator"
"narG, narZ, nxrA; nitrate reductase / nitrite oxidoreductase, alpha subunit [EC:1.7.5.1 1.7.99.-]"
"narT; MFS transporter, NNP family, putative nitrate transporter"
1.000
1.033
1.069
1.107
1.148
1.192
1.240
1.292
1.348
1.409
1.476
1.550
1.632
1.722
1.824
1.938
2.067
2.214
2.385
2.583
2.818
3.100
3.444
3.875
4.429
5.167
6.200
7.750
10.333
15.500
31.000
q-value (corrected)
0.0
15.5
−8000
−6000
−4000
−2000
0
2000
4000
6000
8000
Mean proportion (%)
Difference in mean proportions (%)
